# How a collaborative integrated taxonomic effort has trained new spongiologists and improved knowledge of Martinique Island (French Antilles, eastern Caribbean Sea) marine biodiversity

**DOI:** 10.1371/journal.pone.0173859

**Published:** 2017-03-22

**Authors:** Thierry Pérez, Maria-Cristina Díaz, César Ruiz, Baslavi Cóndor-Luján, Michelle Klautau, Eduardo Hajdu, Gisele Lobo-Hajdu, Sven Zea, Shirley A. Pomponi, Robert W. Thacker, Sophie Carteron, Guillaume Tollu, Adeline Pouget-Cuvelier, Philippe Thélamon, Jean-Philippe Marechal, Olivier P. Thomas, Alexander V. Ereskovsky, Jean Vacelet, Nicole Boury-Esnault

**Affiliations:** 1 Institut Méditerranéen de Biodiversité et d’Ecologie marine et continentale, CNRS, Aix Marseille Univ, IRD, Avignon Univ. Station Marine d’Endoume, chemin de la Batterie des Lions, Marseille, France; 2 Harbor Branch Oceanographic Institute, Florida Atlantic University, Fort Pierce, Florida, United States of America; 3 Instituto de Biologia, Departamento de Zoologia, Universidade Federal do Rio de Janeiro, Rio de Janeiro, Rio de Janeiro, Brazil; 4 Museu Nacional, Departamento de Invertebrados, Universidade Federal do Rio de Janeiro. Quinta da Boa Vista, Rio de Janeiro, Rio de Janeiro, Brazil; 5 Departamento de Genetica, Instituto de Biologia Roberto Alcantara Gomes, Universidade do Estado do Rio de Janeiro, Rua São Francisco Xavier, Rio de Janeiro, Rio de Janeiro, Brazil; 6 Instituto de Estudios en Ciencias del Mar, Universidad Nacional de Colombia, Sede Caribe, c/o INVEMAR. Calle 25 2-55, Rodadero Sur, Playa Salguero, Santa Marta, Colombia; 7 Department of Ecology and Evolution, 650 Life Sciences Building, Stony Brook University, Stony Brook, New York, United States of America; 8 OTEIS. Les Hauts de la Duranne, 370 rue René Descartes, Aix-en-Provence Cedex, France; 9 IMPACT MER. 90, rue du Professeur Raymond Garcin, Route de Didier, Fort-de-France, France; 10 Abyss Plongée. 1 rue des cototiers, Grande Anse, Anses d’Arlet, France; 11 Nova Blue Environment. 14 Rue Chery-Rosette, Fond Lahaye, Schoelcher, France; 12 Marine Biodiscovery, National University of Ireland Galway, School of chemistry, College of Science, Galway, Ireland; 13 Faculty of Biology, Saint-Petersburg State University, 7/9 Universitetskaya emb., St. Petersburg, Russia; University of Genova, ITALY

## Abstract

Although sponges are important components of benthic ecosystems of the Caribbean Sea, their diversity remained poorly investigated in the Lesser Antilles. By organizing a training course in Martinique, we wanted both to promote taxonomy and to provide a first inventory of the sponge diversity on this island. The course was like a naturalist expedition, with a field laboratory and a classroom nearby. Early-career scientists and environmental managers were trained in sponge taxonomy. We gathered unpublished data and conducted an inventory at 13 coastal sites. We explored only shallow water habitats (0–30 m), such as mangroves, reefs or rocky bottoms and underwater caves. According to this study, the sponge fauna of Martinique is currently represented by a minimum of 191 species, 134 of which we could assign species names. One third of the remaining non-identified sponge species we consider to be new to science. Martinique appears very remarkable because of its littoral marine fauna harboring sponge aggregations with high biomass and species diversity dominating over coral species. In mangroves, sponges cover about 10% of the surface of subtidal roots. Several submarine caves are true reservoirs of hidden and insufficiently described sponge diversity. Thanks to this new collaborative effort, the Eastern Caribbean has gained a significant increase of knowledge, with sponge diversity of this area potentially representing 40% of the total in the Caribbean Sea. We thus demonstrated the importance of developing exploratory and educational research in areas historically devoid of biodiversity inventories and systematics studies. Finally, we believe in the necessity to consider not only the number of species but their distribution in space to evaluate their putative contribution to ecosystem services and our willingness to preserve them.

## Introduction

Whereas the concept of ecosystem services is popularized, scientists and environmentalists often confound their perception of biodiversity by neglecting the importance of taxonomy. When considering the different components of biodiversity, the study of species diversity is a time-consuming activity, which can become a considerable challenge for some taxonomic groups, particularly in regions where large gaps of knowledge and lack of expertise exist [1, 2]. In the past two decades, after the emergence of the DNA barcoding concept, naturalists, and even more taxonomists, have developed new concepts and techniques, such as the so-called “integrative taxonomy and systematics”, to maintain or even improve their effort in describing new species or assemblages.

Sponges inhabit all types of aquatic, benthic ecosystems. They can dominate the ocean floors in terms of living biomass and species richness, thus shaping polar, temperate and tropical seascapes [3–5]. In these cases, they are considered keystone components of benthic ecosystem functioning, and powerful suspension feeders, consuming wide ranges of dissolved and particulate organic matter from the pico-, nano- and micro-planktonic communities Depending on the biogeographic region, they experience either anecdotic (temperate seas) or significant predatory pressures (tropical seas) by sea-slugs, fishes and sea-turtles, among other predators [*e*.*g*. 6–8]. In tropical regions, which are usually hotspots of sponge diversity, knowledge of these organisms may be crucial to better understand the putative effects of the global or regional changes, namely climatic anomalies, overfishing or pollution. Sponges are well known to suffer disease outbreaks [9], and are also sometimes considered as key biotic factors threatening endangered reef-building corals [10–12]. In addition, it is also well accepted that sponges provide goods and services with societal benefit. For example, the fact that they are the source of chemical compounds with human health applications stimulates the maintenance of the taxonomic expertise required to describe sponge species diversity. Moreover, their ability to depurate xenobiotics [*e*.*g*. 12–14] further justifies the conservation of sponge communities and highlights the necessity to improve our basic knowledge of their functioning.

The sponge diversity of the Caribbean region has been the subject of intensive studies since the beginning of the 19^th^ century. Lamarck [15] was one of the pioneers of the description of the Antilles’ fauna, but he did not observe specimens in the field, and he gave very few indications about the localities where his studied specimens were collected. Duchassaing and Michelotti were the first taxonomists to actually describe the sponge fauna of the Eastern Caribbean [16, 17]. These authors performed direct observations of living specimens, which allowed them to produce high quality illustrative plates. Although most of the specimens were collected in the British Virgin Islands, some specimens were also collected in Saint-Vincent, Guadeloupe and Vieques.

During the last four decades, many works were published on sponges from the Southern Caribbean [18–22], the Western Caribbean, especially Belize [23, 24] and the Greater Antilles and Bahamas [25–28]. However, the Eastern Caribbean, the Lesser Antilles, and, primarily, the French islands remained poorly investigated [29].

According to the World Porifera Database, about 242 sponge species occurred in the Eastern Caribbean [30]. In the French Antilles, Alcolado & Busutil [31] recently published an inventory of 111 sponge species from Guadeloupe Island, thereby supplementing the few records made by Toffart [32] on epibiont fauna of mangrove roots. Nonetheless, it is noteworthy that only 21 species have been reported from Martinique and almost none from the other French islands (*e*.*g*. Saint Martin, Saint Barthelemy). We can only explain this impressive lack of knowledge in the French Antilles, compared to the Eastern Caribbean area, by the absence of sponge specialists among biologists involved in studies of these islands. We hypothesize that the effort dedicated to Guadeloupe was due to the presence, for years, of a single team of academic marine biologists and to the creation of a Marine National Park, which required fundamental naturalistic studies during the late 1980’s. With the exception of a few episodic publications of new records [29] or new species (*e*.*g*. [33]), the sparse knowledge of Martinique sponge diversity remained within the so-called “grey literature”.

Our main objective in the present work was to fill the gap of knowledge concerning sponge diversity of Martinique. We wanted both to promote the study of sponge taxonomy and to provide a first inventory of the sponge diversity of this island. Our baseline datasets were a few unpublished records of 80–90 species from the CORANTILLES and ECORECIF campaigns (1981 and 1986 respectively; J. Vacelet pers. comm.) and from environmental studies requested by regional agencies in charge of marine environment management (S. Carteron, pers. comm.). We decided to train early-career scientists, students and environmental managers willing to work on sponge biodiversity in tropical seas and especially in French islands. By gathering all data from the grey-literature, the preliminary observations after short field trips and the knowledge acquired during student training, we expected to present a first overall picture of Martinique sponge diversity. To achieve this goal, we organized a training course in sponge taxonomy in December 2013, and we took all participants to the field to confirm the occurrence in Martinique of the 200 most common shallow water species of the Caribbean Sea. After the field course, some students and teachers have continued to study collected sponge samples in their home laboratories according to their biological or geographical relevance.

## Material and methods

### Principles of the Sponge Taxonomy Course (STC)

**Fig 1:**
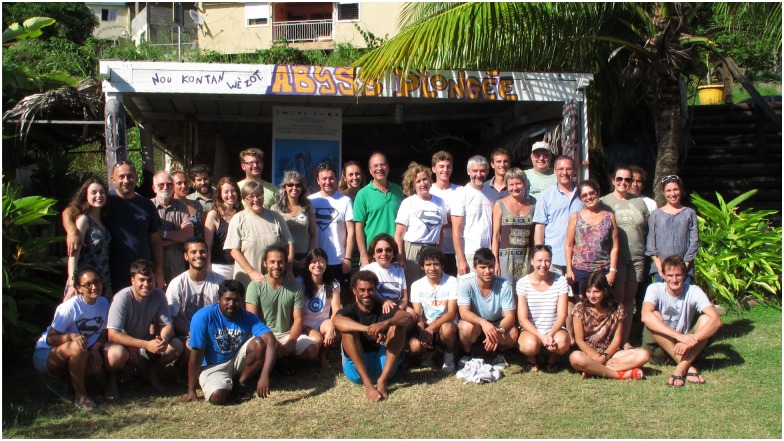
Participants of the Sponge Training Course of Martinique, December 2013. Top, from left to right, S. Griffits, T. Pérez, J. Vacelet, M. Brassy, G. Tollu, E. García, C. Lejeusne, N. Boury-Esnault, M. Klautau, P. Chevaldonné, S. Chenesseau, S. Zea, C. Díaz, J. Chalifour, A. Ereskovsky, E. Tregarot, D. Tokina, E. Hajdu, R.W. Thacker, S. Pomponi, G. Lobo-Hajdu, L. Babarit, A. Pouget-Cuvelier. Bottom, from left to right, B. Cóndor-Luján, H. Fortunato, P. Leocorny, T. Immanuel, C. Ruiz, F. Azevedo, Ph. (Filipo) Thélamon, J. Massei, Z. Hoffman, J. Garcia-Hernandez, M. Łukowiak, A. Sokolova, P-Y. Pascal.

Thirty-five participants attended a training course organized in Anses d’Arlet (Martinique Island) between the 1^st^ and the 8^th^ of December 2013 (Fig 1). All individuals involved gave their written consent to appear in this manuscript. Nine sponge specialists, assisted by three local collaborators, supervised 22 marine biology students, early-career researchers, technicians and environment managers. Our objectives were to provide a fundamental basis of sponge taxonomy, and teach participants how to describe a sponge species, from its external morphology to the examination of its internal morphology (skeleton and even cellular composition), thereby stimulating the careers of new researchers.

**Fig 2:**
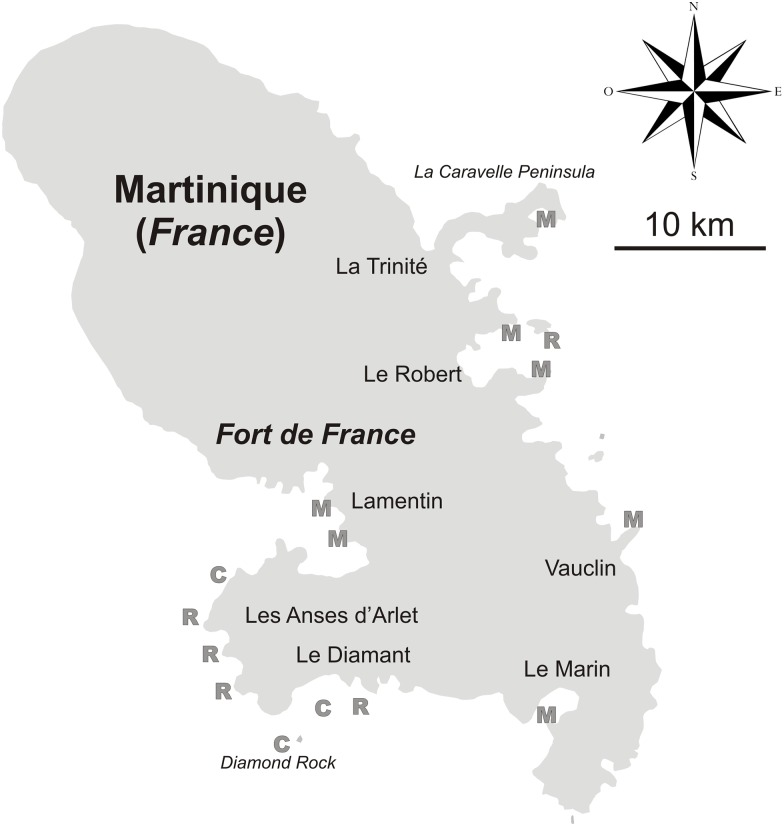
Distribution of the studied sites. C = Cave (dark, semi-dark caves, tunnels, overhangs), M = Mangrove, R = Reef (hard bottoms in general, including coral reefs).

The course combined theoretical lectures, fieldwork and laboratory research. The fieldwork combined snorkeling and scuba-diving observations in different habitats: reefs, mangroves and caves (Fig 2). During five days, every participant went to sea at least once a day to contribute to the overall sampling effort. Participants were asked to apply the same standard sampling protocol, to perform the whole processing of the sample, and then to report their taxonomic work. Most of the taxonomic study was performed in the “sponge camp,” temporarily built for the training course.

### Other initiatives

Several other sampling trips provided data, which remained among the so-called “grey literature” but allowed us to complete our baseline inventory of Martinique sponge diversity.

CORANTILLES and ECORECIF are the oldest cruises organized around the Lesser Antilles. They occurred between 1981 and 1986, organized by the late Professor Jacques Laborel and the University of Antilles-Guyane. These cruises were devoted to diving exploration of the marine environment, with the help of the FFESM (Fédération Française d’Etudes et de Sports Sous-Marins). After CORANTILLES 1 (1981) in Guadeloupe, CORANTILLES 2 (1983–1984) explored Martinique. In 1986, ECORECIF focused on the islands Saint-Barthélémy, Saint-Martin and Anguilla. In Martinique, eight specialists on corals, fishes, marine phanerogams, mollusks, gorgonians and sponges explored 18 localities around the island by scuba-diving. The cruises resulted in several scientific papers and reports [34, 35], and also, especially for CORANTILLES 2 in Martinique, in films and conferences to increase the local awareness of the marine environment.

Between 2010 and 2012, a study on the assessment of the health of Martinique mangroves focused on the epifauna associated with red-mangrove roots, proposing the use of sponges as bioindicators. This study requested by the Regional Direction of the Environment remained unpublished, however three local biologists were trained and then advised by three sponge specialists. The fieldwork was done by snorkeling in mangroves under a gradient of environmental pressures, both along the Caribbean and Atlantic sides of Martinique. Preliminary identifications were done in the field, and a more thorough taxonomic work was achieved in laboratories offering basic facilities for sponge taxonomy.

In the meantime, a small scientific team, composed of 1–3 sponge specialists, began to investigate sponges living in cryptic habitats, such as dark submarine caves, tunnels and overhangs. In these habitats, located between 5 and 22 m depth, the fieldwork was done by scuba-diving. All laboratory work was performed in Marseille, and an integrative taxonomy approach [36], which included metabolomics, genetics, anatomy and morphology, was used.

### Study sites (Fig 2)

Most of the **reefs or rocky bottoms** investigated in this study are on the South Caribbean coast of Martinique, in an area located between Anses d’Arlet and Diamant. The stations explored during the CORANTILLES cruise were about the same as those visited by the students during the STC, ca. 30 years later. They were mostly located around "Grande Anse" (14°29.8’N, 61°5.3’W) and the “Diamond Rock” (14°26.6’N, 61°2.4’W). In these stations, the exploration depths ranged from the surface to 30 m, the bottom being composed of sand, boulders and large rocks in some places. The seascape is dominated by sponges, in terms of diversity and biomass. Sea-fans are also well represented in the shallowest waters, and hermatypic corals are present but not predominant. During the CORANTILLES cruise, a few complementary shallow water dives were also performed off Trois Rivières and Sainte Luce (14°27.9’N, 60°57.2’W), on the Caribbean side of the island, and in the Bay of Robert, on the Atlantic side, around several islets (14°40.7’N, 60°52.8’W).

**Mangroves** are the habitat where the most sampling effort has occurred. Eight different sites were investigated both along the Caribbean and the Atlantic coasts of Martinique, most of them subjected to various sources of anthropogenic pressures. The site “Cohé du Lamentin” (14°36.2’N, 61°01.3’W) is located in a bay head of Fort-de-France, near the mouth of the Lézarde River and in the close vicinity of an industrial area. This mangrove, the second largest in Martinique (350 ha), is subjected to anthropogenic pressure from agricultural, domestic, urban and industrial wastes. The site “Baie de Genipa” (14°33.2’N, 60°59.7’W) is the largest mangrove area in Martinique (950 ha). The watershed is mostly from agricultural and urban areas. “Ilet Baude” (14°26.9’N, 60°52.4’W) and “Pointe Marin” (14°27.0’N, 60°52.9’W) are both located in the outer part of the Marin Bay. This area is subjected to anthropogenic pressures from the City of Marin, where polluting industries and an important harbor are located. On the Atlantic coast, “Grenade” (14°34.0’N, 60°50.0’W) is situated at the entrance of the Cul-de-Sac Petite Grenade, north of Pointe Vauclin. This site is relatively isolated and thus poorly exposed to anthropogenic pressures. “Baie de Saintpée” (14°39.7’N, 60°52.9’W) and “Baie des Requins” (14°41.5’N, 60°54.8’W) are both located in the outer part of the Bay of Robert. A complex watershed, predominantly agricultural, may influence these quite isolated sites. Finally, “Baie du Trésor” (14°46.0’N, 60°53.4’W) is located in the southern part of the peninsula of “La Caravelle”, which is a protected area but nevertheless affected by agricultural and industrial pressures. In these sites, sponges were mainly located on mangrove roots, and occasionally on the muddy sediment. The depth of sampling ranged from the surface to 2 m.

**All cryptic habitats** investigated in this study were located in the south of the island between the cities of Anses d’Arlet and Diamant. The site “Grotte Chauve-Souris” (14°32.0’N, 61°05.3’W) is composed of two close shallow water caves exposed to waves. They are 20–30 m long dark caves, with a maximal depth of 7 m and the bottom composed of little stones and boulders partially covered by hydroids. Near the “Pointe Burgos”, the “Grotte Couleur” is actually a large overhang, 5–10 m deep, where several cave-dwelling marine invertebrates are found. The “Diamond Rock” is crossed by a 50 m long tunnel, 0 to 15 m deep, exposed to waves, and only semi-dark. Between the “Diamond Rock” and the shore, two dark caves, about 100 m distant, are dug into a coral reef formation (so called “cailles”). The “Grotte du Fer à Cheval” (14°28.1’N, 61°01.0’W) has an entrance at 22 m depth, and the “Grotte de Zeb” (14°26.5’ N, 61°03.1’ W) at 17 m depth. They each have a marked gradient of light intensity from the entrance to the rear (10–15 m long) and a certain degree of isolation from the open sea. The bottom of these caves is muddy-sand and a true dark community dominated by sponges covers the walls in some places.

### Sampling and preservation

“Direction de la Mer de la Martinique” provided sampling permits and fully approved this study. Most specimens were photographed before sampling. When this was not possible, a picture of the sample was taken in the laboratory. During the STC, students were given waterproof plates or field guides (S1 Fig) with in situ photographs and keys for identification of 109 species selected among the most common sponges of the Caribbean Sea or previously known from our grey literature references. Students were taught to identify sponges by first identifying species from these underwater plates and then performing complete identifications in the lab (see below). They were then progressively guided in the search for species not on the field guides.

In general, only a fragment of each specimen was collected for identification; the underwater photo and some *in situ* observations enabled the overall description of the specimen shape and color. After collection, samples were kept in seawater for a few hours, and upon return to the laboratory other characteristics were recorded, such as odor, consistency, the putative production of exudates / mucus, or a change in color when exposed to air or ethanol. All samples were preserved in 95% ethanol for morphological study and potential subsequent molecular analyses, and a few samples of species devoid of skeleton were also fixed in buffered 2.5% glutaraldehyde for cytological investigations.

### Skeleton and spicule preparation

During the STC, the protocols to observe the organization of the skeleton and the categories of the spicules were adapted to the conditions of the "sponge camp". For example, to visualize the organization of the skeleton, a piece of each sponge was placed in water in an ice cube tray and frozen in order to prepare a solid specimen from which thin sections could be made with a razor blade, and then observed microscopically.

In normal laboratory conditions, a piece of each sponge is embedded in resin (Araldite^™^) and sections of about 5 mm thick are cut with a low-speed saw and wet-ground with abrasive paper or polishing discs to obtain thinner sections (<1 mm thick), and then mounted on glass slides to study the skeleton architecture microscopically.

Two distinct protocols were applied for the preparation of dissociated spicules. During the STC, the same protocol was applied for Demospongiae and Calcarea. Bleach was used to digest the sponge tissue, and after several rinses, the preparations were placed in a drop of water on slides. Drawings and measurements of spicules were done using compound microscopes. In laboratory conditions, the protocol is different for Demospongiae. A small sponge fragment is boiled in nitric acid until all organic matter is completely degraded. Spicules are then washed several times with distilled water. The resulting suspension is placed on slides for light microscopy or on a metal stubs that are then vacuum coated with gold-palladium for scanning electron microscopy (SEM).

### Complementary investigations

Most of the previous steps in the process of preparing samples for sponge taxonomy can be applied regardless of the availability of laboratory facilities. However, in some cases, a more thorough investigation is needed to record details of spicule morphology or to access new diagnostic characters (e.g. molecular characters). In that case, SEM, histology, cytology or DNA sequencing is performed (for a review see [37]).

In the framework of this study, these complementary investigations were mainly undertaken to eliminate uncertainties, or to provide confirmation when there was a high probability that we had discovered a new sponge species to be described in forthcoming articles. For this reason, about 20% of our records remain identified only at the genus or family levels.

## Results

### Baseline of Martinique sponge diversity

**Fig 3:**
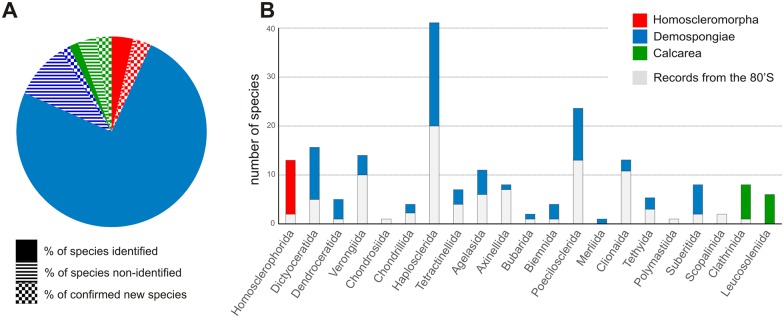
Taxonomic distribution of Martinique sponge diversity.

After analysis of all available data, the sponge fauna of Martinique is composed of a minimum of 191 species (Table 1) of which 134 have been assigned valid species names. This represents an important increase of 173 additional species to the numbers previously known from the World Porifera Database. Three of the four Porifera classes are represented in our list of species: 164 Demospongiae, 13 Homoscleromorpha and 14 Calcarea (Table 1, Fig 3).

**Table 1:** Sponge species recorded in Martinique after the Sponge Training Course in 2013, and comparison with data from the CORANTILLES cruise (1983). C = Cave (dark, semi-dark caves, tunnels, overhangs), M = Mangrove, R = Reef (hard bottoms in general, including coral reefs). Species marked by an * indicate new records for the Eastern Caribbean.

Class	Order	Family	Species	Authorship	1983	2013	Habitat
**Homoscleromorpha**	**Homosclerophorida**	Oscarellidae	*Oscarella* sp. nov. 1	Description in progress by Pérez et al.	X	X	R, C, M
			*Oscarella* sp. nov. 2	Description in progress by Pérez et al.		X	C
			*Oscarella* sp. nov. 3	Description in progress by Ruiz et al.		X	C
		Plakinidae	Aspiculophora madinina*	Ruiz et al. *in press*		X	C
			*Corticium diamantese*	Ereskovsky, Lavrov & Willenz, 2014		x	C
			Plakina arletensis *	Ruiz et al. *in press*		X	C
			*Plakina nathaliae*	(Ereskovsky, Lavrov & Willenz, 2014)		X	C
			*Plakinastrella onkodes*	Uliczka, 1929	X	X	C
			*Plakinastrella* sp.			X	C
			*Plakortis angulospiculatus*	(Carter, 1882)		X	R, M
			Plakortis dariae*	Ereskosvky, Lavrov, Willenz 2014		X	C
			*Plakortis halichondrioides*	(Wilson, 1902)		X	R
			Tetralophophora mesoamericana*	Rützler, Piantoni, Van Soest & Diaz, 2014		X	C
**Demospongiae**	**Dictyoceratida**	Dysideidae	*Dysidea etheria*	de Laubenfels, 1936	X	X	R, M
			*Dysidea janiae*	(Duchassaing & Michelotti, 1864)			R
			*Dysidea* sp.			X	M
		Irciniidae	*Ircinia campana*	(Lamarck, 1814)	X	X	R
			*Ircinia felix*	(Duchassaing & Michelotti, 1864)	X	X	R
			*Ircinia strobilina*	(Lamarck, 1816)	X	X	R
			*Ircinia* sp.			X	R
			Irciniidae sp.			X	R
		Spongiidae	*Hippospongia* sp.			X	R
			*Spongia pertusa*	Hyatt, 1877		X	M
			*Spongia tubulifera*	Lamarck, 1814		X	M
		Thorectidae	Hyrtios cavernosus*	sensu Wiedenmayer, 1977		X	R
			*Hyrtios proteus*	Duchassaing & Michelotti, 1864		X	R
			*Smenospongia aurea*	(Hyatt, 1877)		X	R
			*Smenospongia conulosa*	Pullitzer-Finalli 1986	X	X	R
	**Dendroceratida**	Darwinellidae	Darwinella rosacea*	Hechtel, 1965		X	M
			*Chelonaplysilla* aff. erecta*	(Row, 1911)		X	M
			Chelonaplysilla betinensis*	Zea & Van Soest 1986		X	M
			*Aplysilla* aff. rosea*	(Barrois, 1876)		X	M
		Dictyodendrillidae	*Igernella notabilis*	(Duchassaing & Michelotti, 1864)	X		R
	**Verongiida**	Aplysinidae	*Aiolochroia crassa*	(Hyatt, 1875)	X	X	R
			*Aiolochroia* sp. nov.	Description in progress by Diaz et al.		X	R
			*Aplysina archeri*	(Higgin, 1875)		X	R
			*Aplysina cauliformis*	(Carter, 1882)	X	X	R
			*Aplysina fistularis*	(Pallas, 1766)	X	X	R
			*Aplysina fulva*	(Pallas, 1766)	X	X	R
			*Aplysina insularis*	(Duchassaing & Michelotti, 1864)	X		R
			*Aplysina lacunosa*	(Lamarck, 1814)		X	R
			*Aplysina* sp.		X	X	R
			*Verongula gigantea*	(Hyatt, 1875)		X	R
			*Verongula reiswigi*	Alcolado, 1984	X	X	R
			*Verongula rigida*	(Esper, 1794)	X	X	R
			*Verongula* sp. nov.	Description in progress by Diaz et al.		X	R
		Aplysinellidae	Suberea flavolivescens *	(Hofman & Kielman, 1992)		X	R
	**Chondrosiida**	Chondrosiidae	*Chondrosia* cf. *collectrix*	(Schmidt, 1870)	X	X	R,M
	**Chondrillida**	Chondrillidae	*Chondrilla caribensis* cf. *caribensis*	Rützler, Duran & Piantoni, 2007	X	X	M
			*Chondrilla caribensis* cf. *hermatypica*	Rützler, Duran & Piantoni, 2007		X	R,C
		Halisarcidae	*Halisarca caerulea*	Vacelet & Donadey, 1987	X	X	R,C
	**Haplosclerida**	Callyspongiidae	*Callyspongia fallax*	(Duchassaing & Michelotti, 1864)	X	X	R,M
			*Callyspongia pallida*	Hechtel, 1965		X	M
			*Callyspongia plicifera*	(Lamarck, 1813)	X	X	R
			*Callyspongia vaginalis*	(Lamarck, 1813)	X	X	R
			*Callyspongia* sp.			X	R
		Chalinidae	*Chalinula molitba*	(de Laubenfels, 1949)		X	M
			*Haliclona coerulea*	(Hechtel, 1965)	X	X	M
			*Haliclona implexiformis*	(Hechtel, 1965)		X	M
			*Haliclona manglaris*	Alcolado, 1984		X	M
			Haliclona piscaderaensis*	(van Soest, 1980)		X	M
			*Haliclona tubifera*	(George & Wilson, 1919)		X	M
			*Haliclona vermeuleni*	De Weerdt, 2000		X	M
			*Haliclona vansoesti*	de Weerdt, de Kluijver & Gomez, 1999			R
			*Haliclona curacaoensis*	(van Soest, 1980)		X	M
			*Haliclona smithae*	De Weerdt, 2000		X	M
			*Haliclona* sp. nov.	Description in progress by Diaz et al.		X	M
			*Haliclona* sp.		X		R
		Niphatidae	*Amphimedon complanata*	(Duchassaing, 1850)	X	X	R
			*Amphimedon compressa*	Duchassaing & Michelotti, 1864	X	X	R
			*Amphimedon caribica*	(Pulitzer-Finali, 1986)		X	R
			*Amphimedon erina*	(de Laubenfels, 1936)		X	M
			*Amphimedon viridis*	Duchassaing & Michelotti, 1864	X		R
			Niphates caycedoi*	(Zea and Van Soest 1986)		X	M
			*Niphates digitalis*	(Lamarck, 1814)	X	X	R
			*Niphates erecta*	Duchassaing & Michelotti, 1864	X	X	R
			Niphates alba*	van Soest, 1980		X	R
		Phloeodictyidae	*Siphonodictyon coralliphagum*	Rützler, 1971	X	X	R
			*Siphonodictyon xamaycaense*	Pulitzer-Finali, 1986		X	R
			Siphonodyctium brevitubulatum*	Pang, 1973	X	X	R
			*Calyx podatypa*	(de Laubenfels, 1934)		X	R
			*Oceanapia peltata*	(Schmidt, 1870)	X	X	M
			Oceanapia nodosa*	(George and Wilson,1919)	X	X	R
			Oceanapia bartschi*	(de Laubenfels, 1934)	X		R
		Petrosiidae	*Neopetrosia carbonaria*	(Lamarck, 1814)	X	X	R,C
			*Neopetrosia proxima*	(Duchassaing & Michelotti, 1864)	X	X	R,C
			*Petrosia pellasarca*	(de Laubenfels, 1934)	X	X	C
			Xestospongia arenosa*	van Soest & de Weerdt, 2001		X	R,C
			*Xestospongia muta*	(Schmidt, 1870)	X	X	R
			*Xestospongia* cf. caminata*	Pulitzer-Finali, 1986		X	R
			Xestospongia deweerdtae*	Lehnert & van Soest, 1999		X	R
	**Tetractinellida**	Ancorinidae	*Stelletta* sp.		X	X	R
		Calthropellidae	Pachataxa lutea*	Pullitzer-Finalli, 1986		X	R
		Geodiidae	*Geodia neptuni*	(Sollas, 1886)	X	X	R
			*Geodia* aff. *corticostylifera*	Hajdu, Muricy, Custodio, Russo & Peixinho, 1992	X	X	R
			*Erylus formosus*	Sollas, 1886	X	X	R
		Scleritodermidae	*Aciculites* sp.			X	C
		Tetillidae	*Cinachyrella kuekenthali*	(Uliczka, 1929)		X	R
	**Agelasida**	Agelasidae	Agelas cerebrum*	Assmann, van Soest & Köck, 2001		X	R
			*Agelas citrina*	Gotera & Alcolado, 1987	X	X	R
			*Agelas clathrodes*	(Schmidt, 1870)	X	X	R
			*Agelas conifera*	(Schmidt, 1870)	X	X	R
			*Agelas dispar*	(Duchassaing & Michelotti, 1864)		X	R
			Agelas sventres*	Lehnert & van Soest, 1996	X	X	R
			Agelas tubulata*	Lehnert & van Soest, 1996	X	X	R
			*Agelas* cf. *sceptrum*	(Lamarck, 1815)	X	X	R
			*Agelas* sp.			X	R
		Hymerhabdiidae	Cymbaxinella corrugata (= Axinella corrugata)*	(George & Wilson, 1919)		X	R
			Prosuberites laughlini*	(Diaz, Alvarez & van Soest, 1987)		X	R,M
	**Axinellida**	Raspailiidae	Didiscus oxeata*	Hechtel, 1983	X	X	R,C
			*Didiscus* sp.		X	X	C
			*Ectyoplasia ferox*	(Duchassaing & Michelotti, 1864)	X	X	R
			*Ptilocaulis walpersi*	(Duchassaing & Michelotti, 1864)	X	X	R
		Axinellidae	Axinellidae sp.		X	X	R
			*Dragmacidon reticulatum*	(Ridley & Dendy, 1886)	X	X	R
		Heteroxyidae	*Myrmekioderma gyroderma*	(Alcolado, 1984)	X	X	R
			*Myrmekioderma rea*	(de Laubenfels, 1934)		X	R
	**Bubarida**	Dictyonellidae	Dictyonella arenosa*	(Rützler, 1971)	X	X	R
			*Dictyonella funicularis*	(Rützler, 1971)		X	R
	**Biemnida**	Biemnidae	*Biemna caribea*	Pulitzer-Finali, 1986		X	M
			*Neofibularia nolitangere*	(Duchassaing & Michelotti, 1864)		X	R
			*Neofibularia* aff. *notilangere*	(Duchassaing & Michelotti, 1864)	X	X	R
	**Poecilosclerida**	Acarnidae	*Acarnus radovani*	(Boury-Esnault, 1973)	X		R
		Coelosphaeridae	*Lissodendoryx isodictyalis*	(Carter, 1882)		X	M
			Lissodendoryx spinulosa*	Rützler, Piantoni & Diaz, 2007		X	M
		Crambeidae	*Monanchora arbuscula*	(Duchassaing & Michelotti, 1864)	X	X	R
			*Monanchora* aff. *arbuscula*	(Duchassaing & Michelotti, 1864)		X	R
		Desmacididae	Desmapsamma anchorata*	(Carter, 1882)	X	X	R
		Hymedesmiidae	*Phorbas amaranthus*	(Duchassaing & Michelotti, 1864)	X	X	R
		Iotrochotidae	*Iotrochota birotulata*	(Higgin, 1877)	X	X	R
			*Iotrochota* cf. arenosa*	Rüzler, Maldonado, Piantoni & Riesgo, 2007	X	X	R
			*Iotrochota* sp.		X	X	R
		Microcionidae	*Clathria curacaoensis*	Arndt, 1927	X	X	R
			*Clathria venosa*	(Alcolado, 1984)	X	X	R
			*Clathria* sp.		X		R
		Mycalidae	*Mycale* cf. alagoana*	Cedro, Correia & Hajdu, 2011		X	R
			*Mycale angulosa*	(Duchassaing & Michelotti, 1864)	X	X	M
			Mycale carmigropila*	Hajdu & Rützler, 1998		X	M
			Mycale citrina*	Hajdu & Rützler, 1998		X	M
			*Mycale laevis*	(Carter, 1882)	X	X	R, M
			*Mycale laxissima*	(Duchassaing & Michelotti, 1864)	X	X	R, M
			Mycale magnirhaphidifera*	van Soest, 1984		X	M
			Mycale americana*	van Soest, 1984		X	M
			*Mycale microsigmatosa*	Arndt, 1927		X	M
		Tedaniidae	*Tedania ignis*	(Duchassaing & Michelotti, 1864)	X	X	R
			Tedania klausi*	Wulff, 2006		X	R
	**Merliida**	Merliidae	Merlia deficiens*	Vacelet, 1980		X	M
	**Clionaida**	Clionaidae	*Cliona aprica*	Pang, 1973	X	X	R
			*Cliona delitrix*	Pang, 1973	X	X	R
			*Cliona laticavicola*	Pang, 1973		X	R
			*Cliona tenuis*	Zea & Weil, 2003	X	X	R
			*Cliona varians*	(Duchassaing & Michelotti, 1864)	X	X	R
			*Pione* aff. vastifica*	(Hankock, 1849)	X	X	R
			*Spheciospongia vesparium*	(Lamarck, 1815)	X	X	R
		Placospongiidae	*Placospongia cf*, *intermedia*	Sollas, 1888		X	M
			*Placospongia* spp.		X	X	R
		Spirastrellidae	Diplastrella megastellata*	Hechtel, 1965		X	R
			*Spirastrella coccinea*	(Duchassaing & Michelotti, 1864)	X	X	R,C
			Spirastrella hartmani*	Boury-Esnault, Klautau, Bézac, Wulff & Solé-Cava, 1999	X	X	R,C
			Spirastrella mollis*	Verrill, 1907	X	X	R,M
	**Tethyida**	Tethyidae	Tethya actinia*	de Laubenfels, 1950	X	X	M
			*Tectitethya crypta*	(de Laubenfels, 1949)		X	R
			*Tethya* sp. 1		X	X	R,M
			*Tethya* sp. *2*			X	R
		Timeidae	*Timea* sp.		X	X	R
	**Polymastiida**	Polymastiidae	*Polymastia tenax*	Pulitzer Finali, 1986	X	X	R
	**Suberitida**	Suberitidae	*Aaptos pernucleata*	(Carter, 1870)		X	R
			*Suberites aurantiacus*	(Duchassaing & Michelotti, 1864)			M
			*Terpios fugax*	(Duchassaing & Michelotti, 1864)		X	R,M
			*Terpios manglaris*	Rützler & Smith, 1993		X	M
		Halichondriidae	*Ciocalypta* sp.			X	R
			*Halichondria magniconulosa*	Hechtel, 1965		X	M
			Halichondria melanodocia*	de Laubenfels 1936	X	X	R
			*Hymeniacidon* sp.		X	X	R
	**Scopalinida**	Scopalinidae	*Scopalina ruetzleri*	(Wiedenmayer, 1977)	X	X	R
			*Svenzea zeai*	(Alvarez, van Soest & Rützler, 1998)	X	X	R
**Calcarea**	**Clathrinida**	Clathrinidae	Arthuria hirsuta*	(Klautau & Valentine, 2003)		X	C
			*Arthuria* sp. nov.	Description in progress Cóndor-Luján et al.		X	C
			Clathrina aurea*	Solé-Cava, Klautau, Boury-Esnault, Borojevic & Thorpe, 1991		X	C
			*Clathrina* sp. nov. 1	Description in progress by Azevedo et al.		X	C
			*Clathrina* sp. nov. 2	Description in progress by Azevedo et al.		X	C
			*Clathrina* sp.			X	C
			*Ernstia* sp. nov.	Description in progress Klautau et al.		X	C
	**Leucosolenida**	Amphoriscidae	*Leucilla* sp. nov.	Description in progress Cóndor-Luján et al.		X	C
			*Leucilla* sp. 1			X	C
			*Leucilla* sp. 2			X	C
			*Amphoriscus* sp.			X	C
		Grantiidae	*Leucandra* sp.			X	C
		Leucettidae	Leucetta floridana*	(Haeckel, 1872)	X	X	R,C
		Sycettidae	*Sycon* sp.			X	M

**Fig 4:**
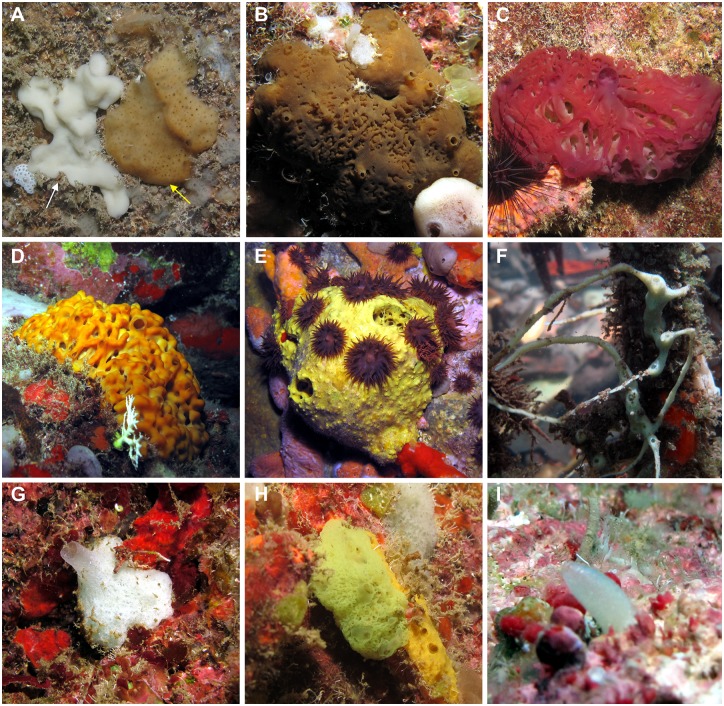
*In situ* photographs of some new records for the Eastern Caribbean Sea. A) *Plakina arletensis* (white arrow) next to the first record of *Tetralophophora mesoamericana* (yellow arrow) in the Eastern Caribbean (“Grotte Chauve Souris”, Anses d’Arlet); B) The aspiculate Plakinidae *Aspiculophora madinina* (“Grotte Fer à Cheval”, Diamant); C, D) Two different *Oscarella* species which are new to science (“Grotte Couleur”, Anse d’Arlet); E) A spherical *Aiolochroia* sp. nov. and its associated sea-anemons (Diamond Rock tunnel); F) A delicately branching *Haliclona* sp. nov. growing on mangrove roots (Bay of Genipa, Lamentin); G) *Leucetta floridina*, a new record for the Eastern Carribean (“Grotte Couleur”, Anses d’Arlet); H) *Ernstia* sp. nov., a new Clathrinidae (“Grotte Couleur”, Anses d’Arlet), I) *Leucilla* sp. nov., a new Amphoriscidae (“Pointe Burgos, Anses d’Arlet). Pictures A,B,C,D,E,G,H,I by T. Pérez, and picture F by C. Díaz.

Thirty-seven taxa were identified only at the genus level, and two at the family level. One third of the unidentified sponge species are definitely considered new to science (Table 2; Fig 4), and their description is in progress in several laboratories. The remaining unidentified species still require more thorough examination, molecular analyses, or even further sampling.

**Table 2:** New sponge species from Martinique which are currently under description.

ID	External traits	Genbank Access numbers	Molecular markers
*Oscarella* sp. nov. 1	Largest *Oscarella* ever recorded. Purple, rarely bright yellow to orange. Thick crust, 2–5 cm, covering surface of up to 1m², fragile consistency, hanging when very large. Lobate surface, large oscules 1–3 cm; often associated with zoanthids.	KX348266	I3-M11 CO1
*Oscarella* sp. nov. 2	Yellow to orange. Thick lobes with oscules from 0.5 to 1 cm. Soft consistency.	KX348267	I3-M11 CO1
*Oscarella* sp. nov. 3	White to gray. Very thin and small crust, with few oscules, smooth surface. Very fragile.	KX348268	I3-M11 CO1
*Plakina arletensis*	White small crust, slightly lobate with a coarse surface and few oscules.	KU674369,1	I3-M11 CO1
*Aspiculophora madinina*	Bright yellow to brown. Massive form, mostly spherical but sometimes pending. Jelly consistency.	KU674367.1	I3-M11 CO1
*Aiolochroia* sp. nov.	Bright yellow, spherical to subspherical with one to three large oscules (2–4 cm). Cavernous interior, thick fibers and cortex		
*Verongula* sp. nov.	Deformed thick tubes, single on in clumps, with one oscule on top of each tube, deep corrugations, and surface usually overgrown by short turf		
*Haliclona* sp. nov.	Small green to gray mounds (<1 cm thick), attached to mangrove roots, with thin (1–2 mm) and long (up to 20 cm) projections profusely extending away from the roots.		
*Clathrina* sp. nov. 1	Light yellow, clathrate with water-collecting tube. Soft.	KX355568—KX355571	Partial 18S, complete ITS-1, 5.8S and ITS-2 and partial 28S.
*Clathrina* sp. nov. 2	Light yellow, clathrate and thin encrusting. Soft.	KX355572	Partial 18S, complete ITS-1, 5.8S and ITS-2 and partial 28S.
*Arthuria* sp. nov.	Yellow, clathrate with water-collecting tube. Soft.		
*Ernstia* sp. nov.	Yellow clathrate. Soft.		
*Leucilla* sp. nov.	Bright white, tubular with apical osculum. Hispid.		

Two Porifera classes, the Homoscleromorpha and Calcarea, have a particularly high number of new species. This increase can be explained by both the investigation of poorly known cryptic habitats, where these two classes predominate, and the contribution of well-trained specialists of these two groups.

Among Demospongiae taxa, the greatest number of species belongs to the order Haplosclerida, with 38 species out of 41 identified at the species level and one species under description; and to the Poecilosclerida, with 22 species out of 24 identified at the species level. Next, the Dictyoceratida and Verongiida are represented by 15 and 14 species, respectively. One new species of *Aiolochroia* and one of *Verongula* are also being described. Polymastiida and Merliida are the two least represented orders, with only one species recorded from each. Among the three dominant orders, two species previously collected in Martinique were not found during our sampling: *Haliclona vansoesti* (Haplosclerida), and *Dysidea janiae* (Dictyoceratida) [19, 38, 39].

During the campaign of 1983, 90 species were collected: 87 Demospongiae, two Homoscleromorpha, and only one Calcarea. Four of the Demospongiae reported by the CORANTILLES were not found during our last sampling; nevertheless, Martinique Porifera diversity doubled after the STC. The improvement of knowledge for Homoscleromorpha and Calcarea is particularly noteworthy, with 11 and 13 species, respectively, added to the diversity of these taxa. The importance of additional sampling is also obvious for some Demospongiae orders such as Haplosclerida with 20 species recorded previously, compared to 41 after the STC, Poecilosclerida with 13 compared to 24 species, Agelasida with 6 compared to 11 species, and finally for the sub-class Keratosa (Dictyoceratida + Dendroceratida) with only six species known previously, compared to 20 species identified after the STC.

### Sponge distribution per habitat

Our sponge inventory is based on the exploration of 13 sites, mostly shallow water habitats (0–30 m), along the SW coast of Martinique, with the exception of the mangroves that were also investigated along the Atlantic side of the island. The sampling effort among these habitats varied greatly, from extensive in mangroves, good in shallow reef and rocky communities, to poor in submarine caves, and some habitats such as seagrass bottoms, deeper coral reefs or deep sea systems, which were not covered at all. However a first trend in sponge distribution between the three main investigated habitats can be described.

The highest species richness values are found in shallow water reefs and rocky bottoms with 127 species taxonomically represented by 97% of Demospongiae from 44 different families. The other two sponge classes are quite rare in these open habitats. In submarine caves, tunnels, and overhangs, 31.5% of the 35 species that have been recorded are Demospongiae from five families, 31.5% are Homoscleromorpha from two families, and 37% are Calcarea from three different families. This type of habitat contains the highest rate of new species, with five Homoscleromorpha and five Calcarea, which are presently under description (Tables 1 and 2). In mangroves, the 49 sponge species recorded are mainly represented by Demospongiae, 46 species belonging to 19 families, two Homoscleromorpha and one Calcarea species.

Surprisingly, of the total of 191 species recorded during the STC, only 20 occur in more than one type of habitat. These species include *Leucetta floridana*, *Mycale laevis*, *M*. *laxissima*, *Oscarella* sp.1 (Fig 4), *Spirastrella coccinea*, *S*. *hartmani*, *S*. *mollis*, *Terpios fugax* and *Tethya* sp. Only the families Spirastrellidae, Chondrillidae and Plakinidae have representatives in all three studied habitats. Therefore, 90% of the sponge species of Martinique have distributions restricted to a specific habitat. This finding is especially the case for various orders of Demospongiae (29 families) and Calcarea (two families), which predominantly occur in reef and rocky bottoms, such as Verongiida, Tetractinellida and Agelasida with respectively 99%, 100% and 99% of their species.

Among Calcarea, all Clathrinida are found in submarine caves, and only one species, *Leucetta floridana*, is also found in the open reef and rocky habitat. All Leucosolenida are also present in caves, with the exception of *Sycon* sp., which is the only Calcarea encountered in mangroves. Only a few species exclusively occur in mangroves: two species of *Lissodendoryx* (Poecilosclerida) and three species of Dendroceratida.

### Main traits of the three types of sponge community

Compared to various other places in the Caribbean region, Martinique is remarkable because of its littoral marine fauna harboring sponge aggregations with high biomass and species diversity. However, on the Atlantic side, macrophytes and hard corals are more dominant, and both the number of sponge species and the size of sponges are reduced.

On coral reefs and rocky substrates along the Caribbean side of the island, from a few meters to deep cliffs, sponges are key components of the seascape. Their biomass is usually greater than that represented by the other common erect sessile organisms, such as gorgonians, antipatharians and hard corals. Some sponge families contribute more to the seascape than others. This is especially the case of the family Aplysinidae, which is represented by 13 species, including two unknown to science, the Niphatidae, and the Agelasidae, which are represented by nine species each.

**Fig 5:**
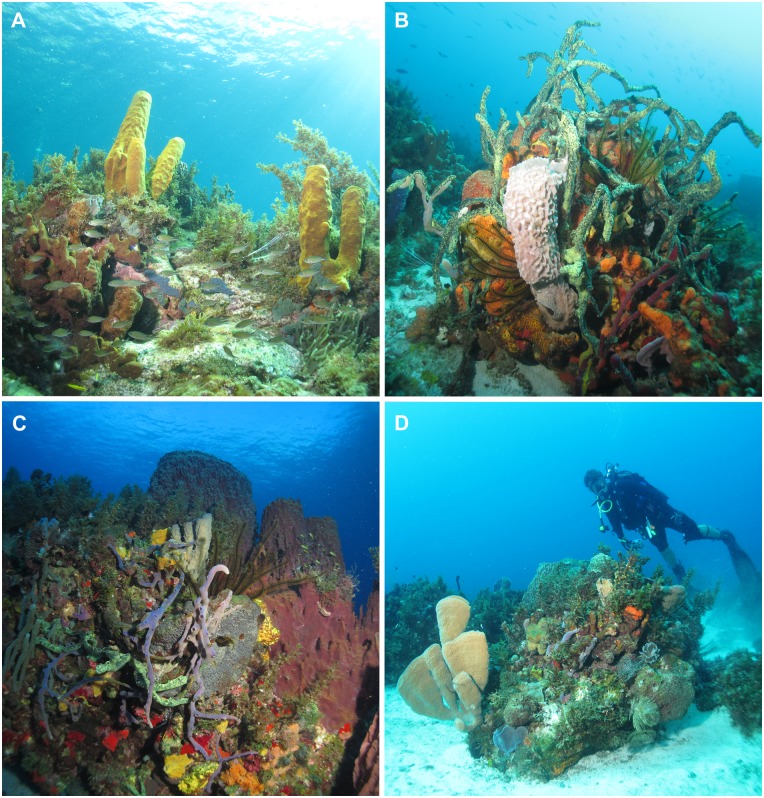
*In situ* photographs of reef and rocky bottoms in Martinique. A) Shallow rocky habitat where tubes of yellow *Aplysina fistularis*, large solid *Agelas* species, and patches of *Halisarca caerulea* mingle between the *Sargassum* sp. algae and the coral rubble. B) Massive sponge aggregations with at least six large sponge species (one large tube of *Callyspongia plicifera*, clumps of *Iotrochota birotulata*, *Agelas* spp., *Aiolochroia crassa*, *Myrmekioderma rea*, *Amphimedon compressa*), and stalk crinoids. C) The largest reef sponges the barrel sponge *Xestospongia muta* aggregates a large diversity of erect and repent species of *Callyspongia*, *Niphates*, *Amphimedon* and *Iotrochota*, massive *Aiolochroia* and *Mycale*, and thin crusts of *Spirastrella*. D) Extremely large tube clumps of *Callyspongia plicifera* reside among the varied set of tubular, rods, and massive shaped sponges that aggregate with polychaete worms, algae, and crinoids. All pictures by T. Pérez.

Specimens of a very large size, sometimes exceeding one meter in height and diameter, were often observed. Examples of such large sponges are the giant barrel sponge *Xestospongia muta* (the most commonly recorded species), the very brightly colored *Callyspongia plicifera*, several species of *Aplysina* and *Verongula*, *Geodia neptuni*, and many others (Fig 5). In the marine area between "Anses d’Arlet" and "Diamant", very large specimens of *X*. *muta*, measuring up to 2 m high, are common; we observed a few individuals of this species that were in various stages of bleaching.

Sponge body sizes and species diversity are reduced in disturbed environments, where, on the contrary, excavating sponges of the genus *Cliona* may develop, with an encrusting shape covering large surfaces. Within our study area, these sites were generally on the borders of the inner zones of the different so-called “Anses”, creeks protected from the waves, well-known for their concentrations of moored sailboats and fishing activities.

Vertical rocky surfaces are colonized by numerous encrusting sponges, generally of bright color (*Spirastrella* spp.), which also contribute to the attractive seascape. On sandy bottoms, where sponges are less abundant, *Oceanapia peltata*, with a spherical body buried in the sediment and visible only by external pagoda-shaped papillae, is found, but only in clean waters. One puzzling observation is related to the rare occurrence of commercial sponges, genera *Spongia* and *Hippospongia*, which are mostly observed in mangroves. There is no report of any kind of past or present sponge harvesting in the French Antilles, whereas organized sponge fisheries exist in several other places in the Caribbean, such as Florida, Cuba, the Bahamas and the Gulf of Mexico.

**Fig 6:**
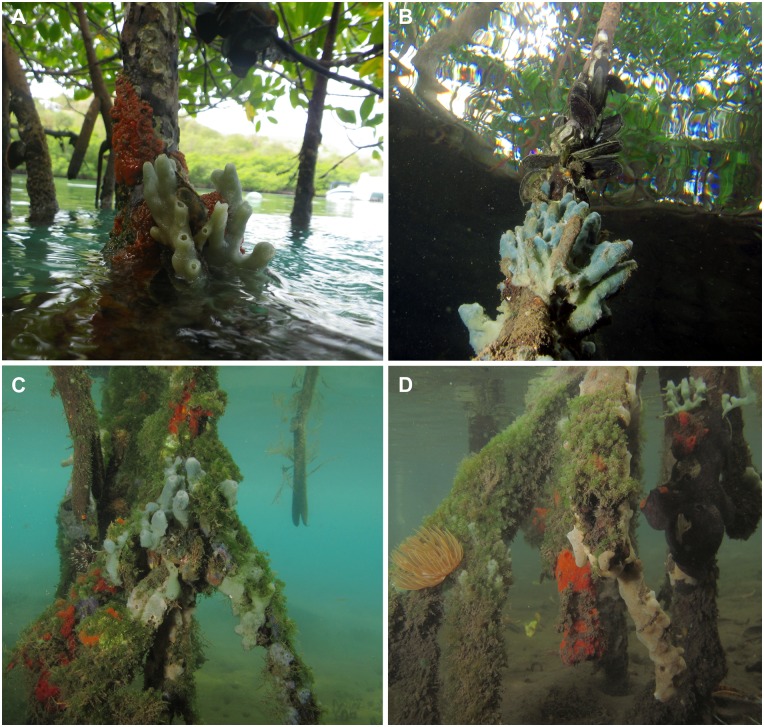
*In situ* photographs of the sponge community growing on mangrove roots in Martinique. A) *Tedania ignis* (the fire sponge) and *Haliclona caerulea* both exposed at low tide. B) Submerged specimen of *Haliclona caerulea*, above a mussel aggregate, and the air exposed zone of the red-mangrove root. C) Green algae (*Caulerpa* sp.), *Haliclona caerulea* and patches of *Tedania ignis*. D) Large root covered by algae, except areas where *Spirastrella mollis* and patches of *Tedania ignis* are seen. Picture A by C. Díaz and pictures B,C,D by T. Pérez.

In Martinique, mangroves are found both on the Caribbean and Atlantic sides, but they are more developed on the southwest of the island. The seaward edge of these mangrove formations is dominated by the red mangrove *Rhizophora mangle*, which grows to moderate height (2–3 m), with roots reaching depths between 30 cm and 1–2 m. On average, sponges cover about 10% of the roots’ surface, which supports a complex biofouling community composed also of cyanobacteria, macrophytes, bivalves, crustaceans, tunicates, and bryozoans. In general, two sponge individuals are found per root, but the community is unstable over time (S. Carteron, pers. obs.). Among the 49 sponge species recorded, two families are dominant both in terms of surface covered and species richness. The family Mycalidae is represented by eight species. *Mycale magnirhaphidifera* can cover up to 100% of the submerged root surface (Fig 6). The family Chalinidae is also represented by eight species, one of them new to science. The new species was found in the “Bay of Genipa” in the shallowest and warmest area (20–30 cm depth) of the red mangrove edge where roots digging into the muddy bottom are usually deprived of macrofauna because of the easy access to predators, such as seastars.

Submarine caves are usually a reservoir of hidden sponge diversity, and because the exploration of the darkest parts of these habitats is not easy, there is always a high potential for new discoveries. Thus far, we have been unable to get any indications of submarine caves on the Atlantic side of Martinique. The northernmost part of the island only has tunnels and overhangs with semi-dark conditions. However, the area between "Anses d’Arlet" and "Diamant" has several cave configurations and a great sponge diversity that remains to be thoroughly investigated.

**Fig 7:**
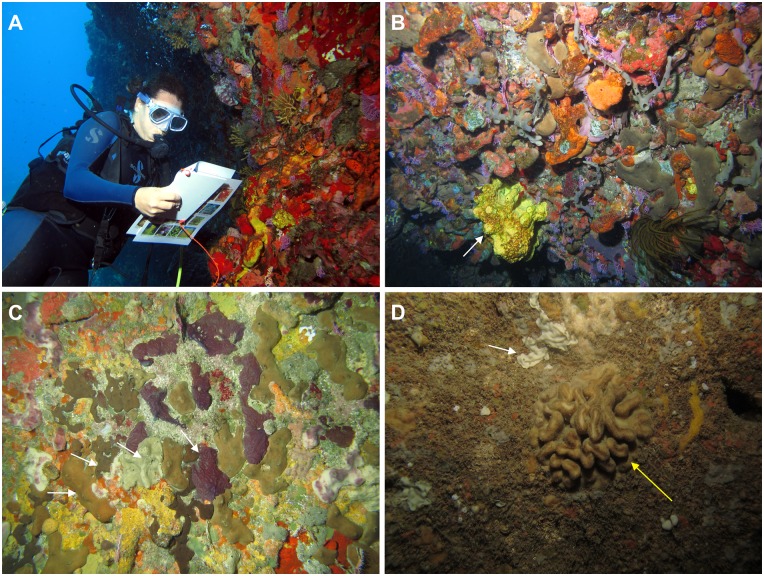
*In situ* photographs of cave community in Martinique. A) A participant of the Sponge Training Course at the entrance of the Diamond Rock Tunnel; B) This semi-dark community dominated by sponges, with the new spherical *Aiolochroia* (white arrow); C) With reduced light, the species diversity decreases, but sponges, especially Homoscleromorpha (white arrows), are still dominant (Diamond Rock tunnel); D) In dark conditions, most sponge species are encrusting forms, such as the new *Plakina arletensis* (white arrow), but in some places big specimens of the lithistid *Aciculites* sp. (yellow arrow) can be found (Grotte Chauve-Souris, Anses d’Arlet). All pictures by T. Pérez.

Diamond Rock is crossed by an impressive 10 to 30 m long tunnel, going from the surface in the middle of the cave to a maximum of 15 m of depth. There is always a strong current and wave action, favoring the development of gorgonians at the entrances and of sponges everywhere, with some walls 100% covered by sponges, including numerous small encrusting specimens (Fig 7). This site still needs a complete sponge inventory. In Anses d’Arlet, there are several shallow water caves (10–30 m long and 3–8 m deep) exposed to the waves, and with a marked light gradient with an associated gradient of sponge diversity from the entrance to the back. The lithistid sponge *Aciculites* sp. (Tetractinellida) was found only in this area. Verongiida are also well represented, but as it was in all cryptic habitats investigated, the high occurrence of unknown Homoscleromorpha is the most striking trait of these systems. This trend is even more pronounced in another cave configuration found in the area. “Grotte du Fer à Cheval” and “Grotte de Zeb” are true caves, dug into the reef, with an average length of 10–15 m long, a depth between 17 and 22 m, a marked light gradient and no detectable wave action, all of which favor the accumulation of mud on the bottom. A first glance into one of these caves revealed up to six unknown species in a single underwater photograph. Two of these species have been already described [33], the thin *Plakina nathaliae* and *Corticium diamantense*, and for the time, we know that Martinique submarine caves that have been explored have at least eight Plakinidae and three Oscarellidae, with four and three new species, respectively, under description. The most remarkable result of the brief exploration of these submarine caves is the discovery of the first aspiculate Plakinidae, *Plakina nathaliae* and *Aspiculophora madinina* [40].

## Discussion

### Revealing a hotspot of sponge diversity in the French Antilles

In the Caribbean Sea, the estimated number of sponge species is about 520 [1], while in the greater Caribbean (including Florida and Bermuda) this number approaches 760 accepted species [3]. The highest values of sponge species richness are from Cuba, with 255 species [1], a number that includes shallow and deep water (> 100 m deep) species. The least diverse Caribbean region appeared to be the Eastern Caribbean, with a total of 126 species recorded. After accounting for important recent works (*e*.*g*. [33, 41]) and an increased effort in incorporating scattered data, the World Porifera Database now contains 242 species for the Eastern Caribbean marine ecoregion [30]. Our inventory of Martinique sponge diversity adds 46 species to this biogeographic region. Once the species new to science are described, the sponge diversity for the region might reach 300 species, representing thus about 40% of the Caribbean sponge diversity. As a result of the present work, gaps of knowledge of the Eastern Caribbean sponge diversity have been significantly reduced, and Martinique can now be compared to other Caribbean areas that have received special attention.

The most important sponge inventory in a French island of the Eastern Caribbean prior to this study is from Alcolado & Busutil [31], who worked in the National Park of Guadeloupe. However, part of the knowledge acquired in Guadeloupe remained in the so-called grey literature. Alcolado & Busutil [31] investigated the reef community and mangroves in only two surveys of one week each, and they then added to their own results some data coming from a report made by Vacelet [42] for the University of Antilles-Guyane. This inventory reached a total of 111 species, 74 species being shared with our present inventory in Martinique. However, 36 species found in Guadeloupe were not found in Martinique, a rather important percentage of sponge diversity that remains to be found, or that might represent true differences between these two islands. In Guadeloupe, Alcolado & Busutil [31] did not consider Calcarea and Homoscleromorpha, so these taxa deserve special attention in future studies. Only one species of the latter was reported, most probably because of the lack of exploration of cryptic habitats. The occurrence of cave-dwelling Homoscleromorpha was also recently confirmed in the northwest of Guadeloupe where a rich sponge diversity also remains to be investigated (T. Pérez and coll., pers. obs.). Nevertheless, the sponge inventories of both islands already share a number of traits. For instance, a total of 18 orders of Demospongiae were recorded in Martinique against 17 in Guadeloupe. In this sponge class, the two most representative orders are the Haplosclerida, with 25 species in Guadeloupe and 41 in Martinique, and Poecilosclerida, with18 and 24 species, respectively. This trait reflects the overall predominance of these two taxa worldwide, with 2314 valid Poecilosclerida and 1114 valid Haplosclerida registered in the World Porifera Database. In both islands, Verongiida, Agelasida and Clionaida were also represented by about 10 species each, which is a usual trait of tropical sponge communities. Regarding the other French islands, Saint Martin and Saint Barthélémy, our knowledge is still too poor to attempt any kind of comparison of sponge diversity patterns.

Interesting trends emerge when we compare Martinique sponge diversity with Curaçao-Bonaire-Aruba, the three Dutch islands of the southern Caribbean where sponge fauna has been intensively studied since 1975 by van Soest and his co-workers [18–20, 43–47]. Van Soest generated his first check-list in 1981, with 166 sponge species distributed among these islands. Taking into account most of the new records coming from this inventory, we estimate that the species richness is about the same as in Martinique. Again, Homoscleromorpha and Calcarea diversities are among the main differences between the two faunas. These results show the importance of having included in the STC experts specialized in these two sponge classes, since most previous sponge biodiversity studies in the Caribbean have reported these two classes as rare taxa, with 1–4 species reported in most Caribbean localities, countries or regions previously surveyed. Among the Demospongiae, some taxa such as the Tetractinellida are much better represented in the Dutch islands sponge fauna, with 21 species recorded as a result of recent surveys conducted in the deep sea ecosystems, down to 500 m depth [47]. Furthermore, the number of Poecilosclerida is presently higher in these islands than in Martinique (33 species vs. 24) probably because of the important research effort of van Soest [20] and also because this group generally requires a more thorough examination of its skeleton (microscleres), which was not possible with the facilities and within the time frame of the STC. Several follow-up studies have just started in our laboratories.

### How to improve our knowledge of sponge diversity in the Caribbean?

The distribution of major taxa among the distinct habitats here studied seemed to indicate a high level of specificity and biased taxonomic diversification within each habitat. For example, Calcarea and Homoscleromorpha seem to have radiated within cryptic habitats shaded from light. On the other hand, for the most abundant Demospongiae, the predominance of certain families on the reef and in the mangroves [48] is here corroborated and extended to other sponge groups. The better examples are the Agelasidae and Aplysinidae on reefs or rocky bottoms, and the Chalinidae and Mycalidae on mangrove roots as many of their species are only found in these habitats. Surprisingly, only one family, Spirastrellidae, was found distributed in all three studied habitats.

At first glance, our study suggests that open habitats on coral reefs and rocky substrates harbor the highest sponge species richness, followed by mangroves and submarine caves. Despite the commonly accepted concept that coral reefs are the richest marine ecosystems, it is also well known that sciaphilous benthic communities harbor the largest numbers of sponge species, sometimes ignored because of their rarity, small size or difficult accessibility [24, 43, 49]. We have just started to explore the hidden diversity of the submarine caves of Martinique, as well as of the Caribbean Sea. Our cave exploration was carried out by only few members of the expedition, at five sites for a total of seven dives. Thus, only two sites were visited twice, and most of the time the sampling focused on two sponge classes, Calcarea and Homoscleromorpha, which are rather rare in other habitats, whereas Demospongiae may represent more than 80% of the sponge diversity in these cryptic habitats. For instance, we have not yet found any Astrocleridae, hypercalcified Agelasida, which were reported in abundance in cryptic habitats of Jamaica [50]. If the level of specificity of the cave fauna is the same in the Caribbean as has been shown in the Mediterranean Sea [51, 52], a more detailed examination of these communities might provide a large number of new records. Such exploration was the objective of a recent campaign organized in 2015 in the Lesser Antilles, which focused on littoral dark habitats (PACOTILLES cruise). The new biological material from this campaign is currently being analyzed. A better understanding of the deeper sea ecosystems is no longer disconnected from the societal economic issues related to the coastal zones. Deep sea ecosystems and submarine caves share a large number of traits, especially their low resilience to environmental disturbances and the connectivity patterns between the coastal and the deep sea zones [53]. In the French Antilles, only few collections have been taken from deep water habitats, and the sponge material that has been collected remains to be studied (Pomponi unpublished data.)

Sponge communities, particularly on reefs, tend to be dominated in terms of biomass by only a few species, such as *Agelas* spp., *Aplysina* spp., *Callyspongia* spp., *Niphates* spp., and *Xestospongia muta*, with the largest proportion of species present in low abundance and with a fragmented distribution. This trait was already reported in other Caribbean reefs and mangroves [54], as we observed during the STC campaign. Therefore, we suggest that sponge biodiversity surveys must involve experts of various taxa in several locations and large areas (*e*.*g*. hundreds of meters of mangrove shoreline) to increase the chances of encountering less abundant species and, consequently, the largest number of species.

Taxonomic surveys might focus on particular functional groups within sponges. For example, large erect species create heterogeneity in the form of complex three-dimensional habitats that can host fisheries-targeted mobile fauna (*e*.*g*. fishes and lobsters). Species harboring photosynthetic microsymbionts contribute to the carbon pump, whereas excavating sponges, such as Clionidae, bore into substrates and make carbon bioavailable. Both categories can be abundant on the open reef, but their abundance and dominance depends on environmental health, which again is the result of the degree of anthropogenic pressures.

### The benefit of improving taxonomic capabilities

The significant increase of sponge diversity knowledge acquired through this work is directly related to the sampling effort that was deployed and the readily available expertise. An important accomplishment of the STC was its organization in a region lacking any technical and scientific infrastructure, despite the fact that the site was in the vicinity of environments with diverse marine life. The STC was set up like a naturalist expedition, with a field laboratory and a classroom nearby, which facilitated training a large percentage of participants who were true beginners (14 out of 22) in sponge biology (Table 3). The only prerequisite expected of attendants of the course was a fundamental knowledge of organismic biology or marine ecology. A minimum level of scuba diving expertise was expected for participation in all types of sampling, however, every day, half of the sampling effort was made by just snorkeling. Participants were selected according to their professional ambition in the Caribbean Sea (research, education and management of the marine environment) or in other tropical regions (Pacific and Indian Oceans). All were able to reproduce several times all the steps necessary for a biodiversity inventory, and each has completed at least one formal description of a sponge species. At the end of the STC, they were all trained to use the proper terminology for describing the external and internal morphological features that allow identifying specific sponge groups. Thus, although true specialized taxonomists could not be trained in the time-frame of the STC, all participants were provided with tool-kits to launch their own taxonomic analyses and to better communicate and exchange information within a community of sponge experts.

**Table 3:** List of the students and young researchers who attended the Sponge Training Course in December 2013. FR France, BR, Brazil, CO, Colombia, IN, India, ME, Mexico, PE, Peru, PL, Poland, PR, Puerto Rico, RU, Russia, UK, United Kingdom,. PRO = professional, EDU = education, EX = private environmental expertise / consulting, SC = Scientist, AP = Assistant Professor, RA = Research Associate, RC = Researcher, GR = Graduate student, MS = Master’s student, PhD = Phd student, PDoc = Post-doctoral fellow, TECH = technician or engineer, MPA = Marine Protected Area. S+: previous experience with sponges; S-: no experience, beginner working with sponges.

Nom	Country	Status during STC	Current status
AZEVEDO Fernanda	BR	SC, PDoc, S+	PDoc, working on calcareous sponges molecular taxonomy, biodiversity and phylogeography at the Federal University of Rio de Janeiro, Brazil
BRASSY Mathilde	FR	PRO, EDU, S-	Running educational activities, has implemented a sponge culture on artificial reefs, at Carbet des Sciences, Martinique, France
CHALIFOUR Julien	FR	PRO, MPA, S-	Scientific Officer / leader for the MPA of Saint Martin, France
CHENESSEAU Sandrine	FR	SC, TECH, S-	Technician working on sponge histology and cytology, CNRS, Marseille, France
CHEVALDONNE Pierre	FR	SC, RC, S+	Researcher collaborating on sponge molecular taxonomy, CNRS, Marseille, France
CONDOR LUJAN Baslavi	PE	SC, PhD, S+	PhD candidate on calcareous sponges biodiversity and connectivity at the Federal University of Rio de Janeiro, Brazil
FERRY Romain	FR	PRO, EDU, S-	PhD candidate on Martinique marine species diversity, including a sponge inventory, University of Antilles, France
FOLCHER Eric	FR	SC, TECH, S-	Technician at IRD in New Caledonia, contributing to sponge samplings and inventories, Nouméa, France
FORTUNATO Humberto	BR	SC, MS, S+	PhD candidate studying on sponge integrative taxonomy at the State University of Rio de Janeiro, Brazil
GARCIA HERNANDEZ Jaaziel	PR	SC, MS, S-	Master candidate at the University of Puerto-Rico-Mayaguez
GARCIA-BONILLA Erika	CO	SC, PhD, S-	PhD Candidate studying sponge microbiology
GRIFFITS Sarah	UK	SC, MS, S-	PhD candidate studying o sponge microbiology and population genetics at the University of Manchester, United Kingdom
HOFFMAN Zvi	ME	SC, MS, S-	Master candidate at the Autonomous University of Southern Baja California, La Paz, Mexico, and applying for PhD in the USA
IMMANUEL Titus	IN	SC, RA, S+	PhD candidate studying sponge biodiversity of the Andaman Islands, India
LEJEUSNE Christophe	FR	SC, PDoc, S-	AP at the University Pierre et Marie Curie, partly working on sponge biodiversity in French Brittany, Roscoff, France
LEOCORNY Pedro	BR	SC, GR, S+	MS candidate studying sponge population genetics and phylogeography at the Federal University of Rio de Janeiro, Brazil
ŁUKOWIAK Magdalena	PL	SC, PDoc, S+	Assistant Professor mainly working on spicular analysis and reconstructing fossil sponge association at the Institute of Paleobiology of the Polish Academy of Science, Poland
PASCAL Pierre-Yves	FR	SC, AP, S-	Assistant Professor at University of Antilles, teaching sponges and conducting research projects on sponge ecology, Guadeloupe, France
RUIZ Cesar	CO	SC, PhD, S+	PhD candidate studying sponge biodiversity in submarine caves, Aix Marseille University, France
SOKOLOVA Agniya	RU	SC, MS, S-	Joining a PhD program on sponge ecology at the University of Saint Petersburg, Russia
TOLLU Guillaume	FR	PRO, EX, S-	Leading environmental and marine biodiversity studies, Impact Mer, Martinique, France
TREGAROT Ewan	FR	PRO, EX, S-	Environmental and marine biodiversity studies in general, OMMM, Martinique, France

From the student-participant point of view, the intellectual atmosphere, made up of internationally recognized experts in their field, was particularly appreciated. The unique concentration of specialists, a good student/teacher ratio and accessibility, and the availability of all the organizers were also among the main strengths identified after completion of the STC. Today, a great majority of the former STC student-participants are working on sponges, in various contexts but mostly running their own research projects (Table 3). They are often working in locations where gaps of knowledge exist (*e*.*g*. Brazil to the Caribbean Sea, India), studying patterns of diversity (species, population genetics, phylogeography), deciphering species-complexes, or applying the integrative taxonomy approach. As a result of a novel trend in environmental sciences, some of the participants are applying holistic approaches such as metagenomics or metabolomics.

From the teacher-participant point of view, exchanging ideas with early-career scientists about their research projects and, on a daily basis, looking anew at sponge biodiversity was highly stimulating. Linking together experts in various taxonomic groups with well-trained Caribbean naturalists has been a great benefit of the STC experience. This improvement of scientific communication is obvious for our experience in Martinique where a knowledge gap existed, and this experience should also be applied to islands where sponge inventories have been already done. Increasing the number and frequency of such workshops would allow participants to target particular sponge groups, revealing a challenging taxonomy, or dedicating a homogenous effort to poorly investigated habitats harboring a rich diversity. Another successful example of this approach was a series of Sponge Taxonomy Training Courses and workshops carried out at the Smithsonian Tropical Research Institute in Bocas del Toro, Panama, over the past ten years, which revealed one of the most diverse localities for Caribbean biodiversity of shallow water sponges (C. Diaz and R. Thacker pers. comm.).

The present study provides baseline data about French Antilles sponge diversity that will contribute to the development of marine protected areas and conservation corridors. The collaboration allowed catalyzing efforts to move from a list of species that was previously available in the grey literature, about 40% of the present knowledge, to an extensive inventory of shallow water sponge communities, including sponges associated with mangroves and underwater caves. The STC contributed 30% of the present knowledge of Martinique sponge diversity, and, all together, brought our expertise to the current level of the rest of the eastern Caribbean. Currently, the open reef and rocky habitats seem the most diverse in Martinique, but we expect that a major source of unique species still awaits discovery, particularly under rocks, and under the roofs and crevices of caverns and caves.

Several vocations arose among the STC participants who were not truly established in sponge taxonomy, and the Martinique community is now more aware of the usefulness of sponges in a global assessment of ecosystem functioning. There are already a large number of on-going research projects and some peer-reviewed publications that will feed global databases, such as the World Porifera Database.

The main outcome from this collaborative work is the demonstration of the importance of developing exploratory, educational and biodiversity research in areas historically devoid of inventories and systematics studies. A large number of reports refers to the Caribbean islands as “biodiversity hotspots”. Our collaborative effort has placed a new hotspot on the map of Atlantic Biodiversity.

## Supporting information

S1 Fig Waterproof plates presenting *in situ* pictures together with visual keys of identification of the most common sponges of the Caribbean Sea. (PDF)
