# Supplementary material for: How a collaborative integrated taxonomic effort has trained new spongiologists and improved knowledge of Martinique Island (French Antilles, eastern Caribbean Sea) marine biodiversity
Source: PLoS One. 2017 Mar 22;12(3):e0173859. doi: 10.1371/journal.pone.0173859 (PMC5362083; doi:10.1371/journal.pone.0173859)
Supplement: S1 Fig — (PDF) [file pone.0173859.s001.pdf]

THE CARIBBEAN SPONGE DIVERSITY
Reef (1)

Pictures : Thierry Pérez, Cristina Diaz, Michelle Klautau, Sven Zea, Bob Thacker, Sophie Carteron, Guillaume Tollu, Eduardo Hajdu and cortesy of Guilherme Muricy, Leandro Monteiro, Klaus Ruetzler and Martin Kammer

CALCAREA Class
Calcaronea Subclass

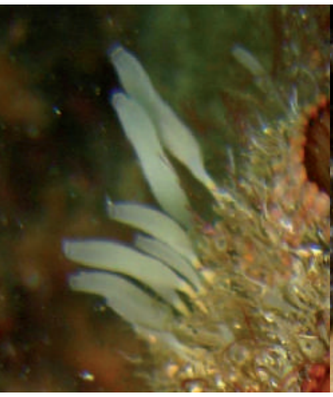

***Amphoriscus* sp. nov.**
vase, friable, oscules 2 mm

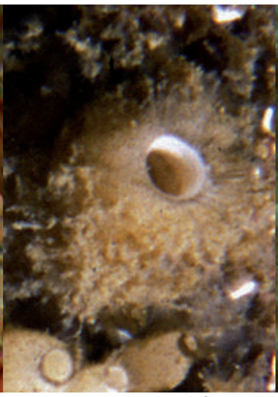

Leucandra rudifera
vase, crowned, oscules 3 mm

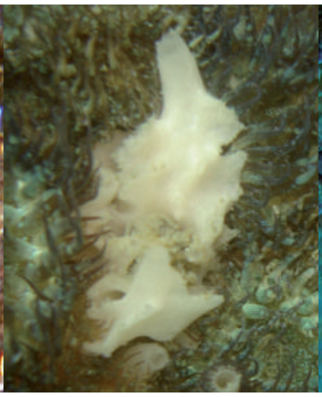

***Leucilla* sp. nov.**
massive amorphous, friable, oscules 5 mm

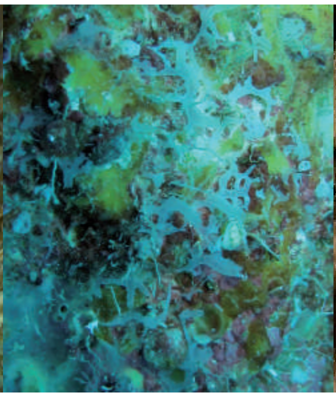

***Leucosolenia* sp.**
tubes protruding from a stolon not anastomosing, oscules 1 mm

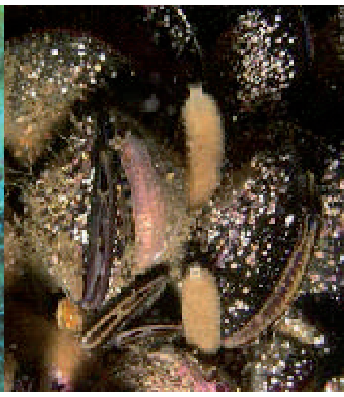

***Sycon* sp. nov.**
vase, friable, oscules 1-2 mm

Calcinea Subclass

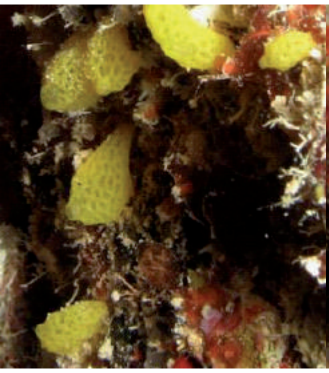

***Clathrina* sp. nov.**
tubular anastomosed, yellow, soft, 1-2 cm wide

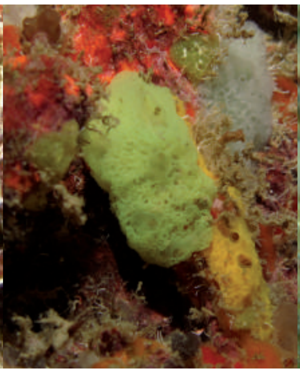

***Clathrina* sp. nov.**
tubular anastomosed, light yellow, soft, 1-3 cm wide

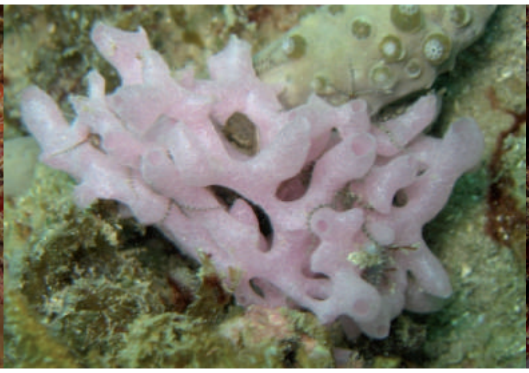

Leucaltis clathria
tubular, white to pink, friable, tubes 4-6 mm wide

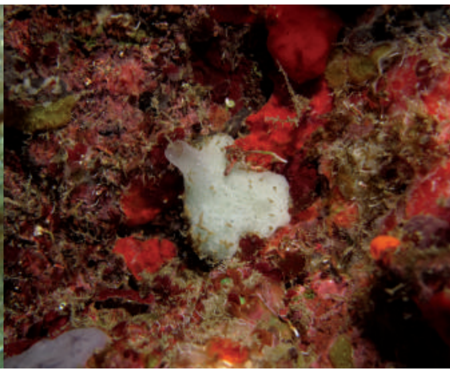

Leucetta floridana
massive, white to blue, friable, oscules 1-4 cm

HOMOSCLEROMORPHA Class

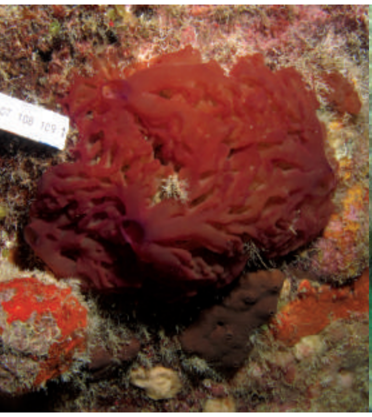

***Oscarella* sp.**
thin crust, lobate, oscules 0.5-1 cm

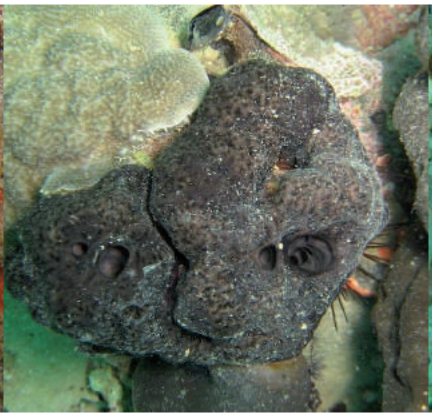

Plakinastrella onkodes
massive to tubular, gray brown to black, hard, oscules 1-2 cm

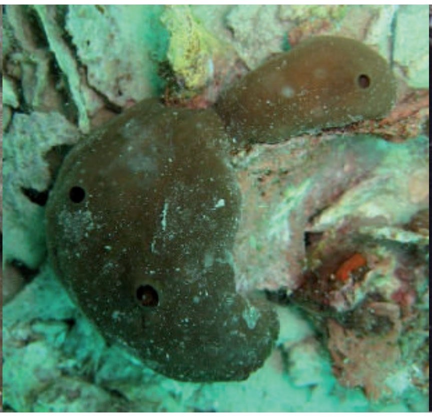

Plakortis angulospiculatus
smooth and dense, brown to gray, oscules < 1 cm

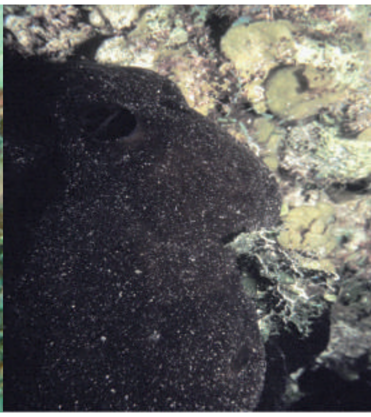

Plakortis halichondrioides
smooth, dark brown to black, dark exhudate

DEMOSPONGIAE Class
Agelasida Order

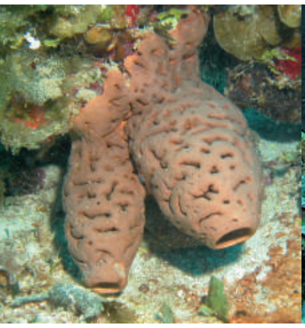

Agelas cerebrum
brown, grooved barrel-tubes (5-15 cm wide, 20-40 cm long), oscules on top of tubes

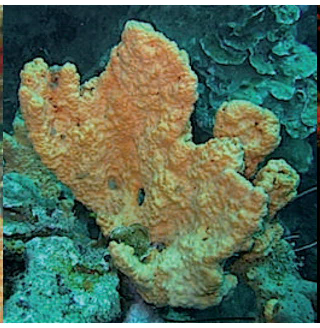

Agelas citrina
ear-shaped, golden brown to orange, sparse round oscules 3-5 mm

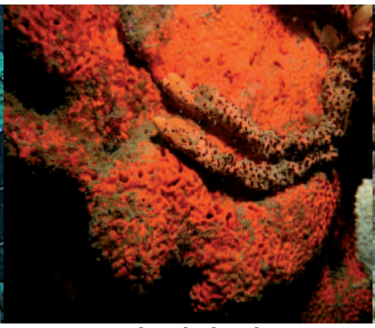

Agelas clathrodes
orange and flabellate (back) diverse oscules
A. sceptrum erect branches (front) oscules 2-3 mm with round membrane

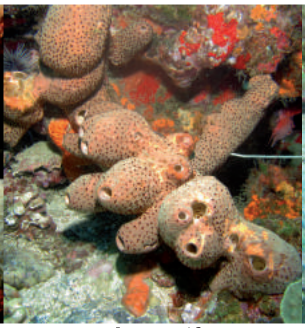

Agelas conifera
orange brown, smooth tubes (5-15 cm wide, 30-60 cm long), oscules on top of tubes

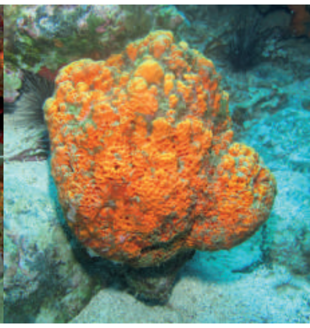

Agelas dispar
brown sepia flabellate or massive, round oscules 2-4 mm, larger key hole deformed openings

THE CARIBBEAN SPONGE DIVERSITY
Reef (2)

Pictures : Thierry Pérez, Cristina Diaz, Michelle Klautau, Sven Zea, Bob Thacker, Sophie Carteron, Guillaume Tollu, Eduardo Hajdu and cortesy of Guilherme Muricy, Leandro Monteiro, Klaus Ruetzler and Martin Kammer

Axinellida Order

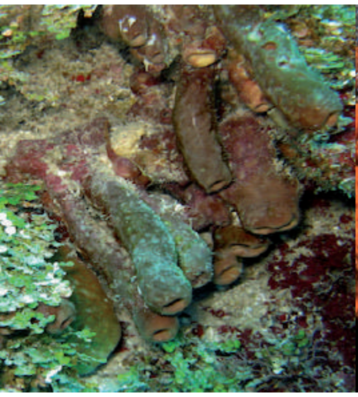

Agelas wiedenmayeri
cluster of tubes 2-3 cm wide 6-20 cm long, oscules on top and on side of tubes, round to key-holed

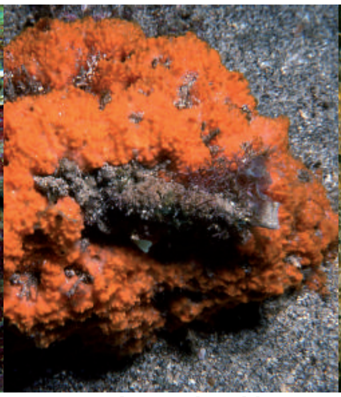

Prosuberites laughlini
crustose, orange, rugose surface, oscules 1-6 mm with membrane

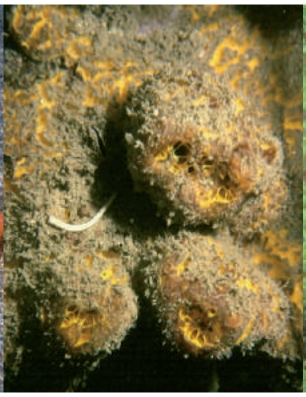

Didiscus oxeata
massive clumps, yellow-gray, meandering grooves at surface 1-5 mm wide

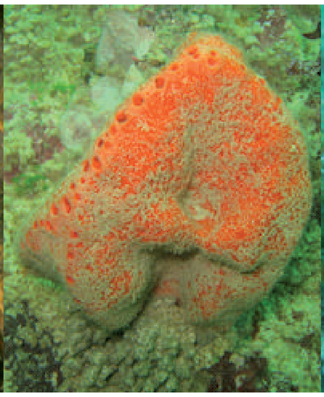

Dragmacidon reticulatum
red thick crusts to mounds, conulose (5 mm), oscules 3-6 mm wide

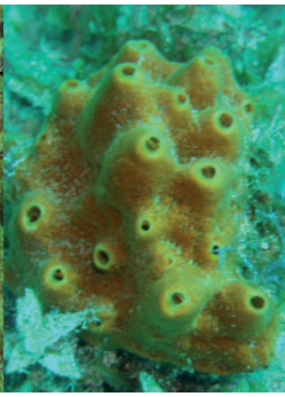

Ectyoplasia ferox
brown-orange massive to lobate, oscules 2-10 mm, with brown rim and slightly elevated

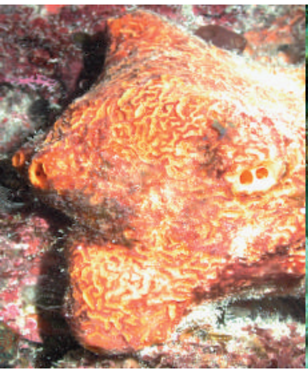

Myrmekioderma gyroderma
massive lobate, orange-yellow, meandering grooves at surface, oscules 1-2 cm

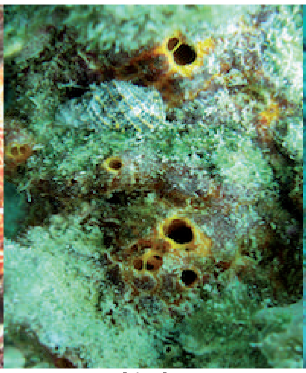

Myrmekioderma rea
crust filling crevices, orange-yellow, circular grooves at surface, oscules 1-3 cm

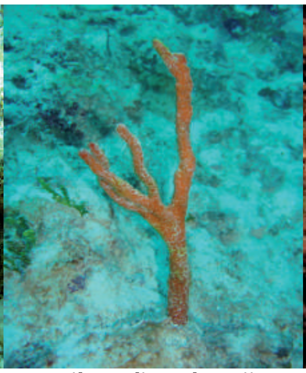

Ptilocaulis walpersii
erect thin (1-3 cm) branches, orange red, surface with spatulate projections

Chondrillida Order

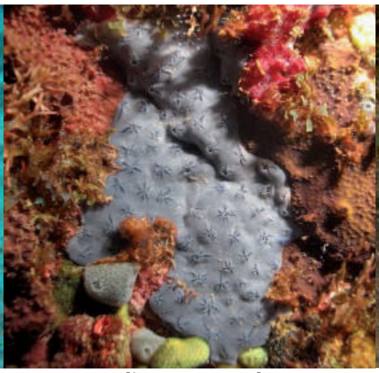

Halisarca caerulea
thin smooth and slippery crust, star-shaped oscules 2-3 mm

Chondrosiida Order

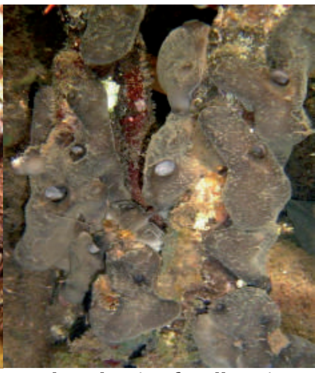

Chondrosia cf. collectrix
brown to black, massive to crust, cartilage consistency, oscules 1-4 mm wide

Clionaida Order

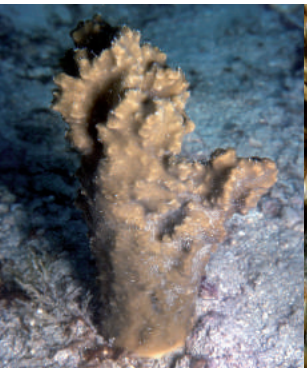

Cervicornia cuspidifera
antler like, hollow, deformed tips, cream-brown, tough

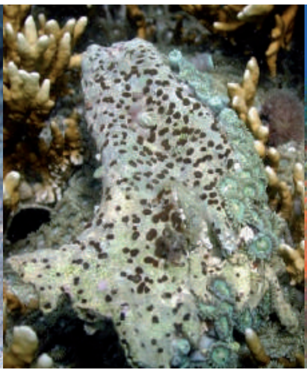

Cliona aprica
dark brown, small papilla 1-3 mm wide, sponge tissue within coral frame

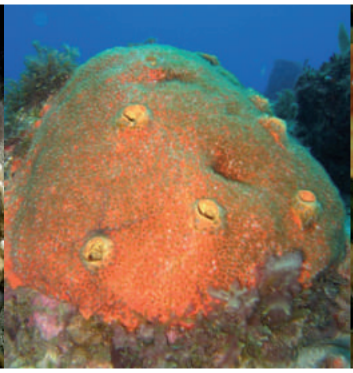

Cliona delitrix
crustose, boring, orange to red, round papilla 2-5 mm, oscules with membrane (1-3 cm wide)

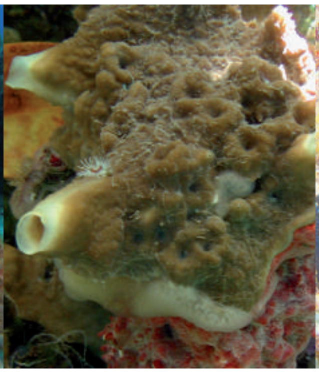

Cliona varians
crust to massive , brown greenish, brown membranaceous oscules 2-30 mm wide

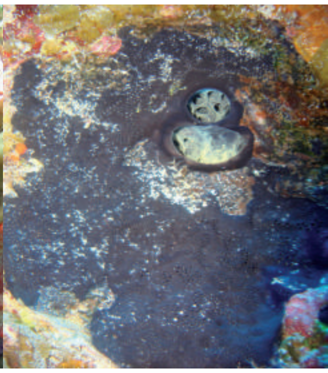

Spheciospongia vesparium
black, leathery crust to globular, large oscules 1-5 cm

Dictyoceratida Order

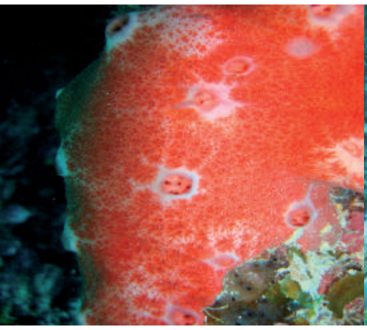

Spirastrella hartmani
rustose, salmon to dull orange, membranaceous whitish oscules 5-20 mm wide

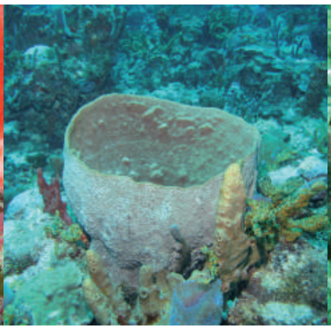

Ircinia campana
thin walled vase, oscules 1-5 mm, inner side, dificult to tear

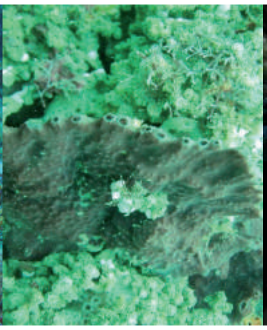

Ircinia felix
variable shape amorphous to turret shaped, conules 1-2 mm, oscules 4-8 mm

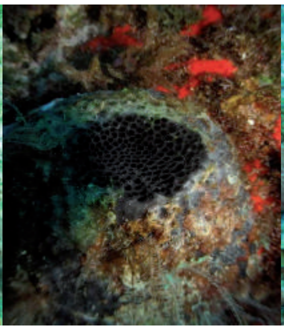

Ircinia strobilina
massive, globular, conules 5 mm high, oscules 2-15 mm, aggregated on top

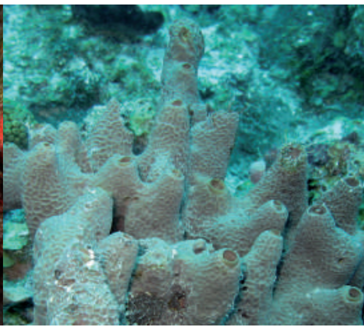

Smenospongia aurea
massive-lobate, yellow to brown, honey-combed surface, soft-firm, mucosy when cut

THE CARIBBEAN SPONGE DIVERSITY
Reef (3)

Pictures : Thierry Pérez, Cristina Diaz, Michelle Klautau, Sven Zea, Bob Thacker, Sophie Carteron, Guillaume Tollu, Eduardo Hajdu and cortesy of Guilherme Muricy, Leandro Monteiro, Klaus Ruetzler and Martin Kammer

Haplosclerida Order

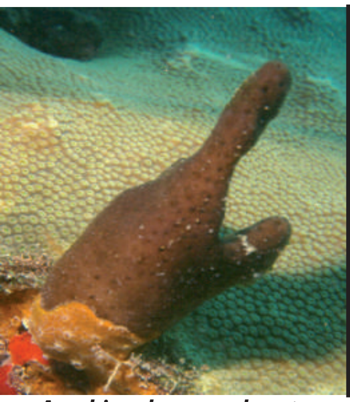

Amphimedon complanata
massive to flabelliform, dark brown-purple to black, oscules 1-3 mm wide

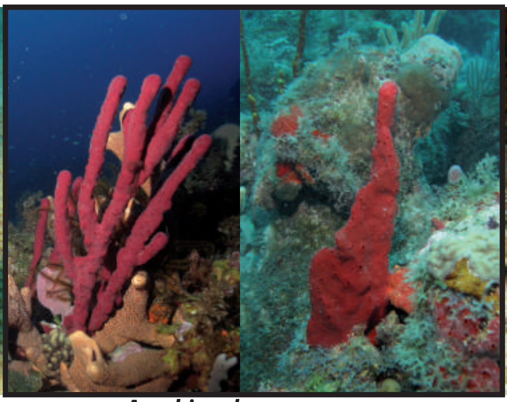

Amphimedon compressa
red ramose to flabelliform or massive, oscules 2-8 mm wide

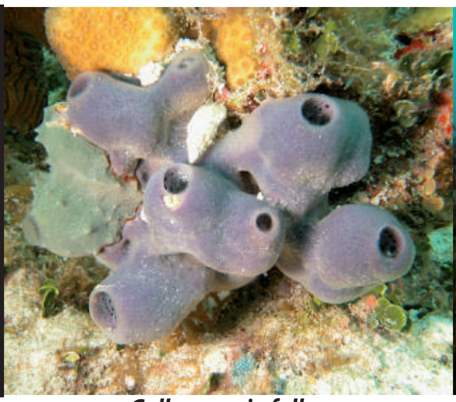

Callyspongia fallax
short tubes to branches (3-4 cm wide), cream-gray to purplish, smooth, oscules 1-2 cm wide

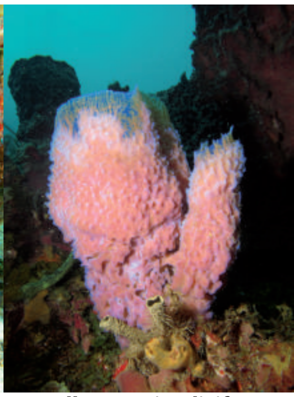

Callyspongia plicifera
vase, outer surface with meandering grooves, iridescent pink, bluish, oscules 1-3 mm

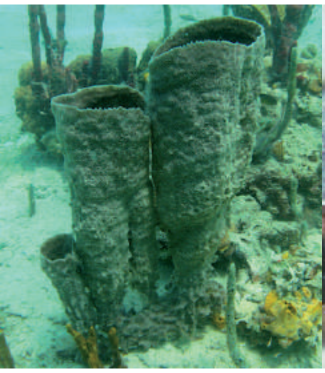

Callyspongia vaginalis
gray to pinkish, vase or tubes with 5-10 mm thick walls, conules 3 mm high, 5 mm apart

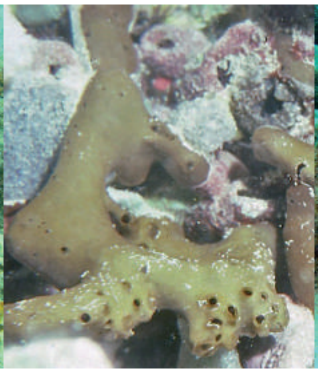

Calyx podatypa
fragile, hollow massive to branching , smooth, riddled by apertures < 1 cm wide

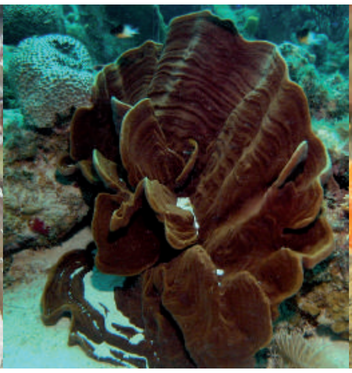

Cribrochalina vasculum
single to anastomosing plates, 1-2 cm thick, velvety surface, reddish brown

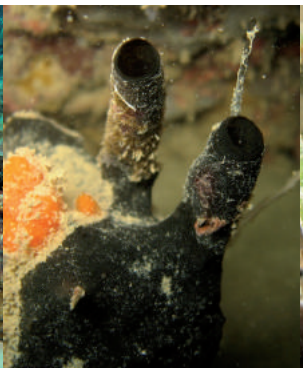

Neopetrosia carbonaria
repent branches to massive, hard, black and hollow, oscules 3-6 mm wide

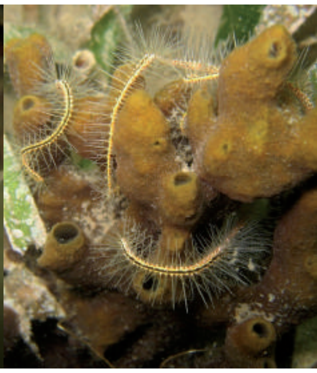

Neopetrosia proxima
massive, lobate, thick crust, brown to burgundy, oscules 3-10 mm

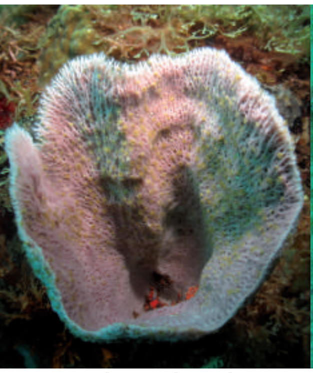

Niphates digitalis
gray, pink to purplish, oscules inner wall, hispid surface between conules (1-3 mm high)

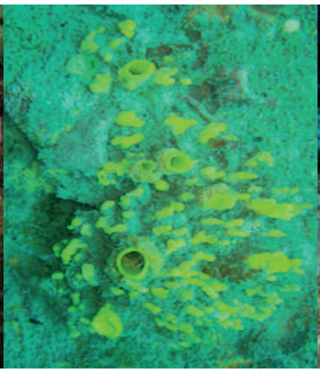

Spongosorites coralliophaga
excavating, yellow, oscular tubes 1-3 cm wide

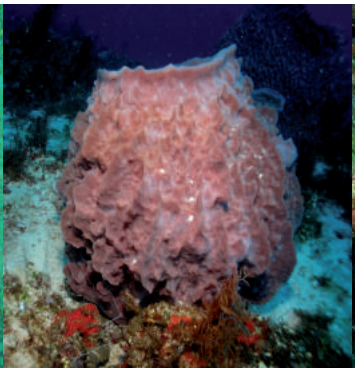

Xestospongia muta
barrel sponge, red to burgundy, oscules 1-3 mm on inner wall

Poecilosclerida Order

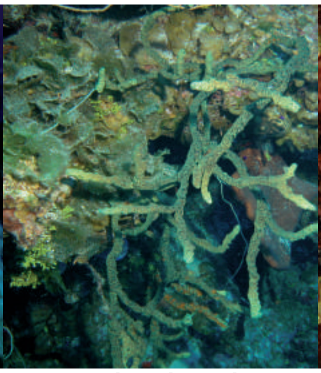

Iotrochota birotulata
sprawling branches, dark green to black, spiky, oscules 1-3 mm

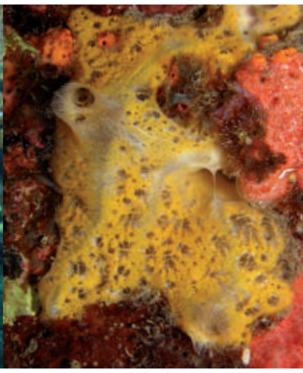

Mycale laevis
thick crust to branching, orange to white, oscules 1-4 cm with white strands on membrane

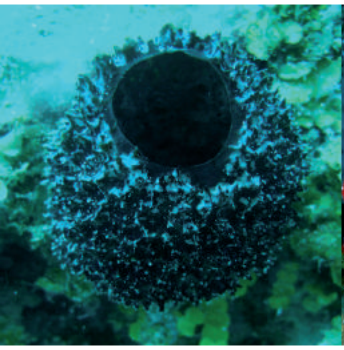

Mycale laxissima
tubular to globular, dark red to black, wall 1-2 cm, spiky pseudo-oscule 3-6 cm

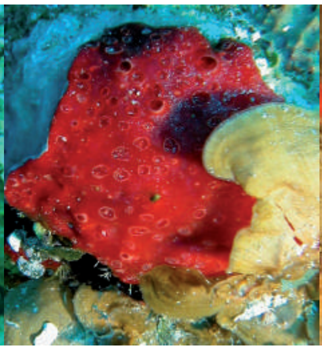

Phorbas amaranthus
bright red crust, areolated surface, oscules 5-20 mm

Polymastiida Order

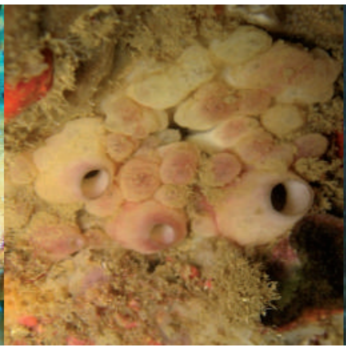

Polymastia tenax
brown to red-brown mounds with papilla, oscular chimneys (5-30 mm wide, 10-20 mm high)

Scopalinida Order

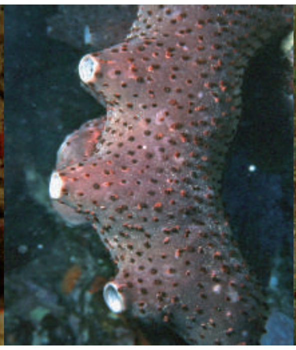

Svenzea zeai
massive to ramose, velvety, soft, oscules 1-2 cm wide

Suberitida Order

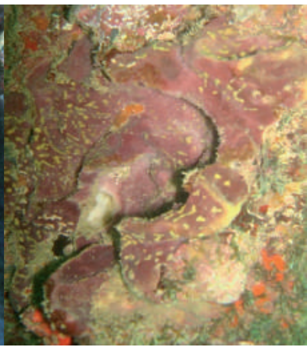

Topsentia ophiraphidites
massive to platy, hard, smooth-velvety surcafe, red-brown to white

THE CARIBBEAN SPONGE DIVERSITY
Reef (4)

Pictures : Thierry Pérez, Cristina Diaz, Michelle Klautau, Sven Zea, Bob Thacker, Sophie Carteron, Guillaume Tollu, Eduardo Hajdu and cortesy of Guilherme Muricy, Leandro Monteiro, Klaus Ruetzler and Martin Kammer

Tetractinellida Order

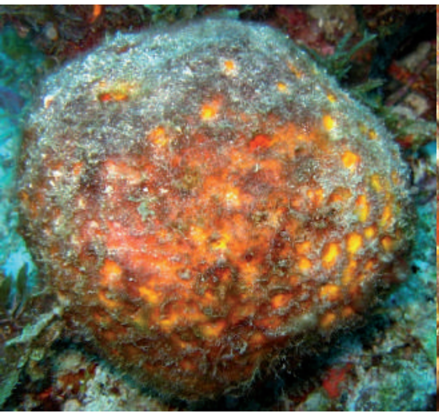

Cinachyrella kuekenthali
round, up to 20 cm wide, orange redish to gray, porocalices 5 mm

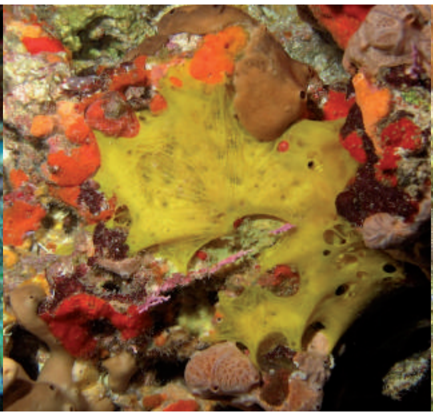

Dercitus luteus
thin yellow crust, smooth and leathery

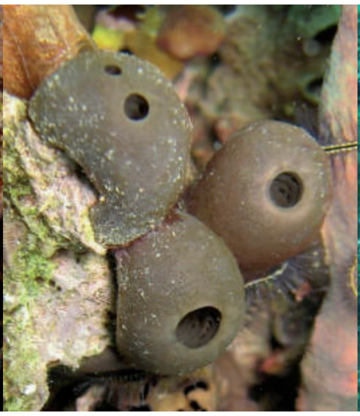

Erylus formosus
massive cushions, brown to black, smooth, oscules 1-10 mm

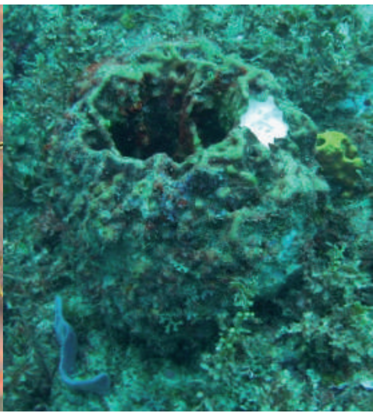

Geodia neptuni
massive to globular, greenish brown, rugose and hard, oscules on top plate few mm wide

Verongiida Order

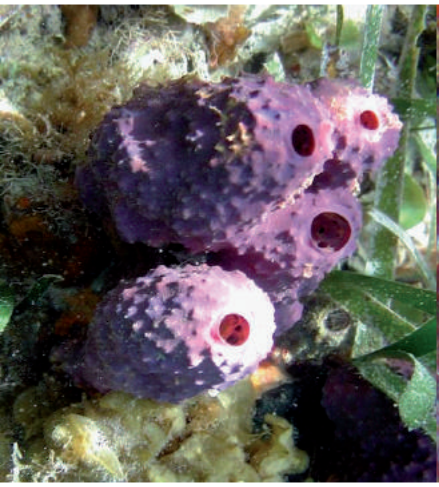

Aiolochroia crassa
bright yellow to purple, lobes or masses, round conules, oscules 1-2 cm

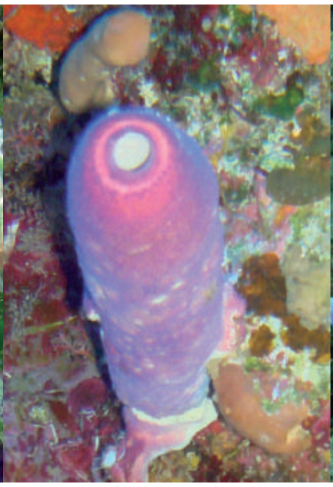

Aplysina archeri
purple to violet tubes, micro-conulose (< 1 mm high), firm

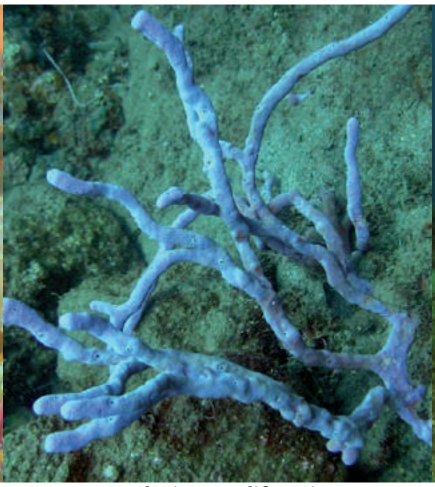

Aplysina cauliformis
pink to purple-brown, branches, conules 0.1 mm high

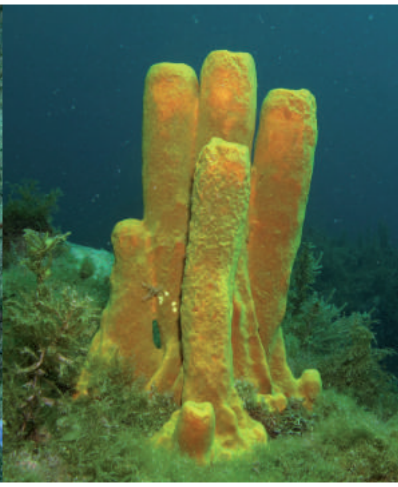

Aplysina fistularis
yellow tubes, > 10 cm high, 2-8 cm wide, conules 5 mm

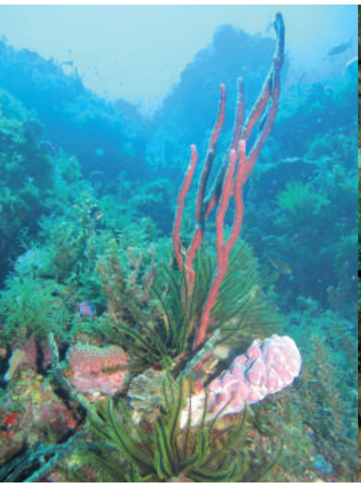

Aplysina fulva
yellow to ochre branches, conules 0.5 mm

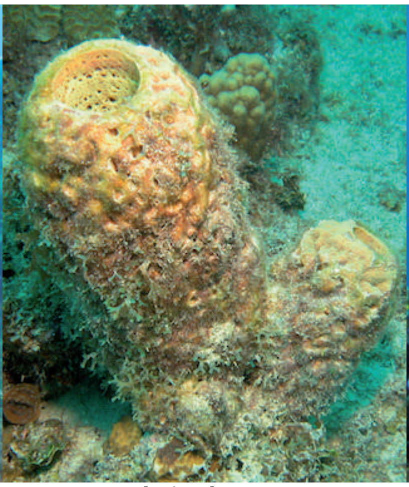

Aplysina lacunosa
yellow-brown tubes, with strongly grooved surface, groves up to 1 cm wide

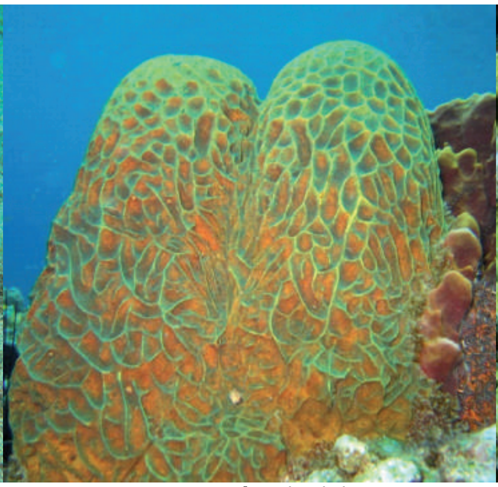

Verongula reiswigi
yellow green, tubes up to 30 cm high, ridged surface, soft consistency, oscular membrane

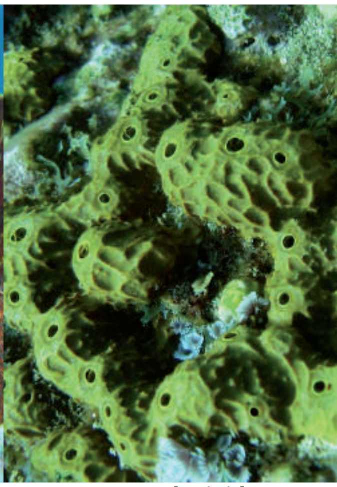

Verongula rigida
yellow to brown lobes,"honey comb" surface, firm, oscules 0.5-1.5 cm wide, with membrane

THE CARIBBEAN SPONGE DIVERSITY
Mangrove (1)

Pictures : Thierry Pérez, Cristina Diaz, Michelle Klautau, Sven Zea, Bob Thacker, Sophie Carteron, Guillaume Tollu, Eduardo Hajdu and cortesy of Guilherme Muricy, Leandro Monteiro, Klaus Ruetzler and Martin Kammer

DEMOSPONGIAE Class

Biemnida Order

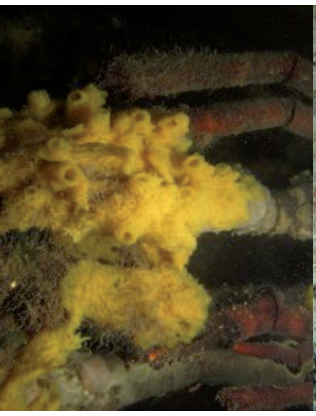

Biemna caribea
massive crustose, yellow, soft,
oscules 1-4 mm wide with membrane

Chondrillida Order

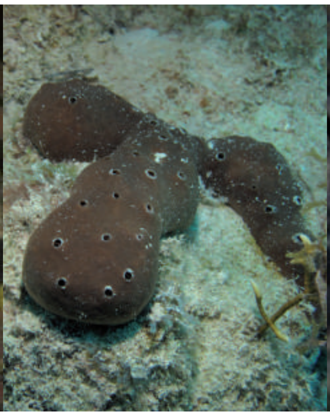

Chondrilla caribensis
crust to massive, gray to dark brown,
smooth, oscules 1-2 mm

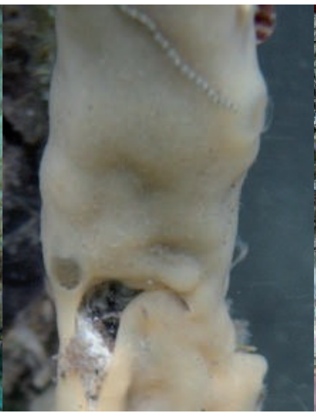

Halisarca restigaensis
thin crust, cream-yellowish,
slippery, oscules 1-2 mm

Dendroceratida Order

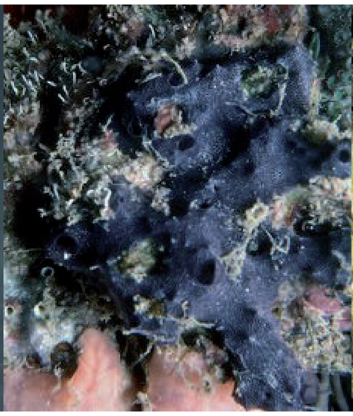

Chelonaplysilla aff. erecta
spiky, very soft, black, conules 3-5 mm

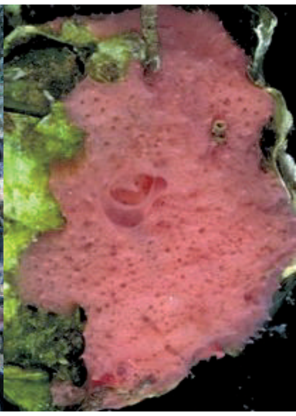

Darwinella rosacea
thin crust and porous, pink or rose,
soft, conules 0.5 mm

Dictyoceratida Order

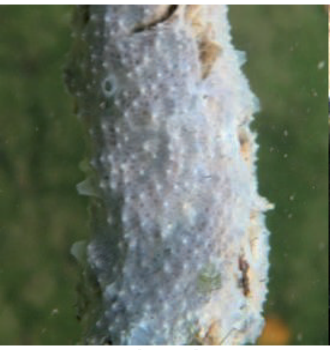

Dysidea etheria
crust or massive, soft, blue pink,
conules 1 mm high, 2-4 mm apart

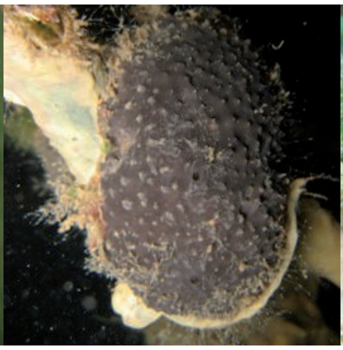

Hyrtios proteus
massive, compressible, black out and
brown in, conules > 1 mm

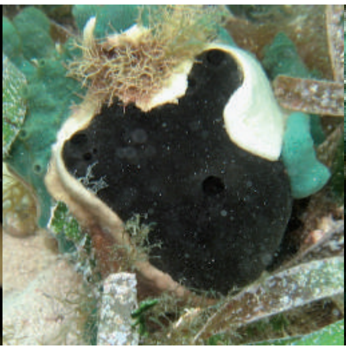

Spongia pertusa
massive, compressible and visually smooth,
black out and brown in, conules < 5 mm

Haplosclerida Order

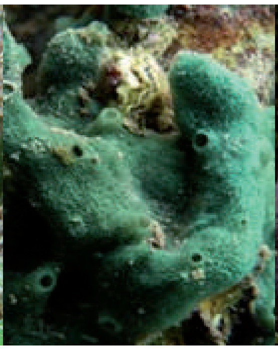

Amphimedon erina
crust to lobate, firm, oscules 2-4 mm

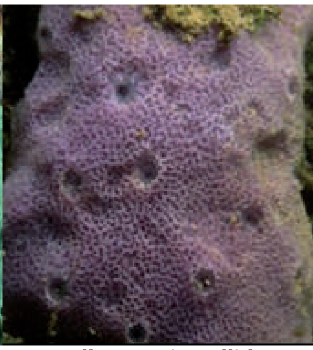

Callyspongia pallida
crust and porous, firm, violet to brown,
oscules 2-4 mm with white membrane

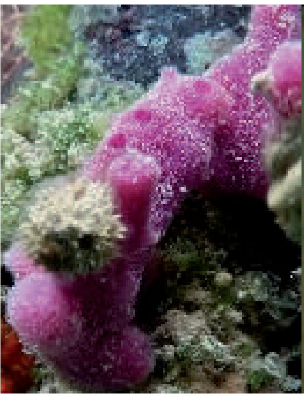

Chalinula molitba
cushions to ramose, flimsy,
oscules 1-7 mm

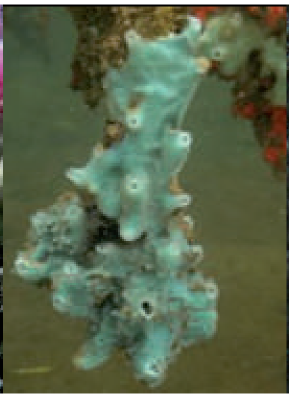

Haliclona caerulea
crusts cushions or digitate, bluish,
firm, oscules 1-8 mm

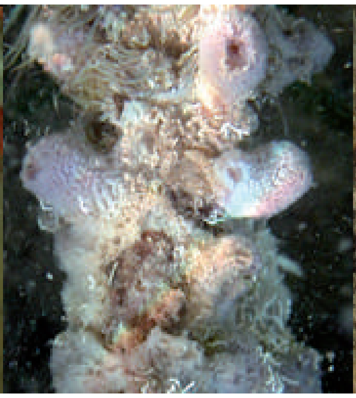

Haliclona curacaoensis
crust to cushions, gray to whitish, rugose
compressible sticky mucous, oscules 5-15 mm

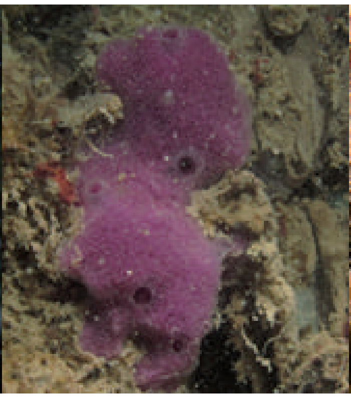

Haliclona implexiformis
crust to cushions, rose to violet,
compressible, oscules 4-10 mm

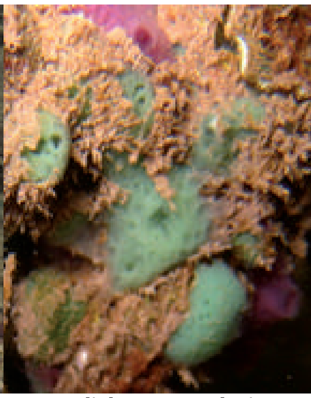

Haliclona manglaris
thin crust to cushions, soft, oscules 1-3 mm

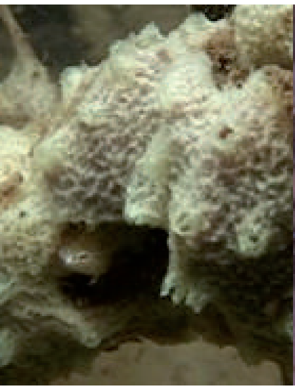

Haliclona cf. piscaderaensis
crust, friable, grayish white,
oscules 2-3 mm

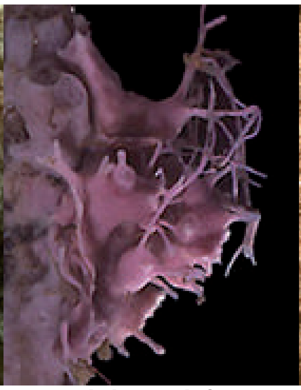

Haliclona tubifera
crust to cushion, pink to violet,
firm, with filaments

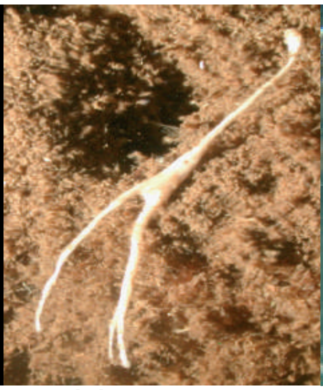

Haliclona twincayensis
dichotomous thin (1-3 mm)
branches, oscules < 1 mm

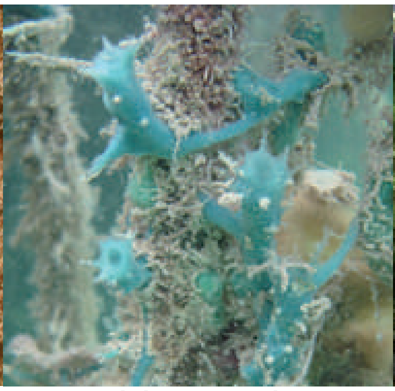

Haliclona vermeuleni
tubes with spiky extensions, turquoise
to gray, firm, oscules 1-3 mm

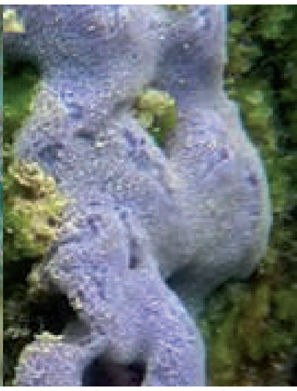

Niphates caycedoi
crust, violet to pink, firm, oscules 4-8 mm

THE CARIBBEAN SPONGE DIVERSITY
Mangrove (2)

Pictures : Thierry Pérez, Cristina Diaz, Michelle Klautau, Sven Zea, Bob Thacker, Sophie Carteron, Guillaume Tollu, Eduardo Hajdu and cortesy of Guilherme Muricy, Leandro Monteiro, Klaus Ruetzler and Martin Kammer

Poecilosclerida Order

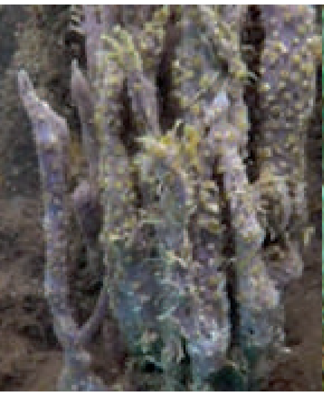

Niphates erecta
massive to ramose, rose to gray, hispid surface, oscules 3-7 mm

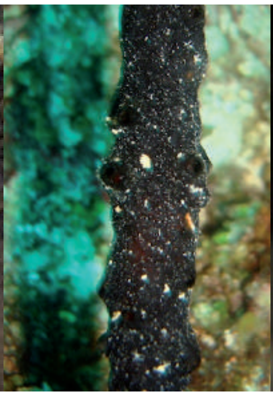

Artemisina melana
crustose, dark brown out and orange in, soft, oscules 5-10 mm

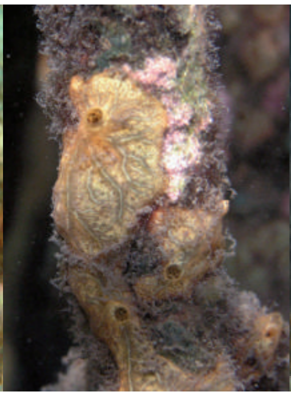

Clathria curacaoensis
crust to ramose, orange to red with yellow tinges, oscules 4-10 mm and can be as small as 1 mm

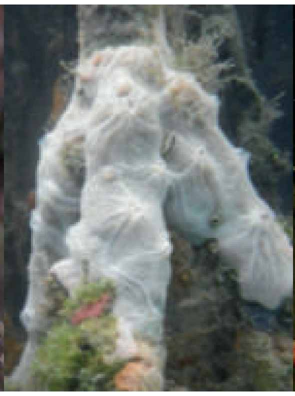

Clathria venosa
thin crust, gray to cream, oscules 2-4 mm, with radial canals

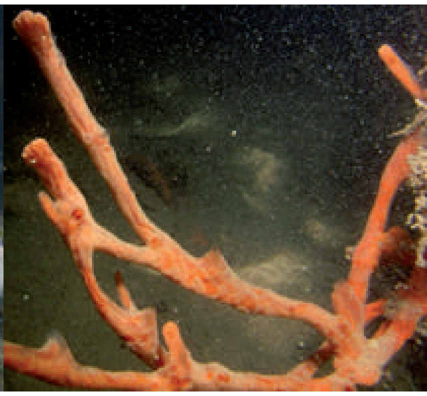

Desmapsamma anchorata
lobate to ramose, pink-red to orange, oscules 1-5 mm with membrane

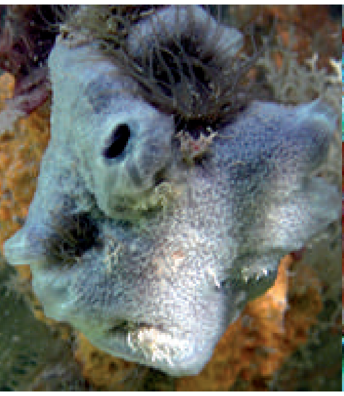

Lissodendoryx isodictyalis
massive to lobate, pale gray green violet, oscules 4-10 mm, with membrane and one side canal

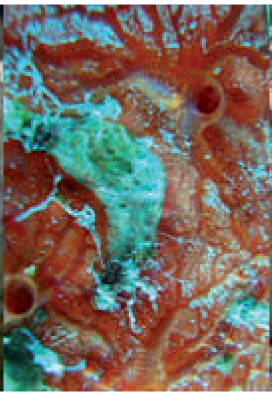

Monanchora arbuscula
crust to ramose, red with transparent membrane, oscules 3-20 mm

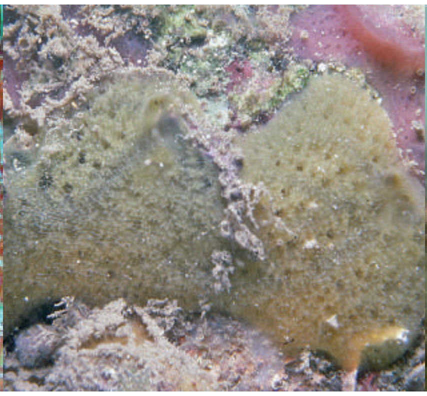

Mycale americana
crust 0.5-20 mm, yellow to orange, mucose, oscules 3-10 mm

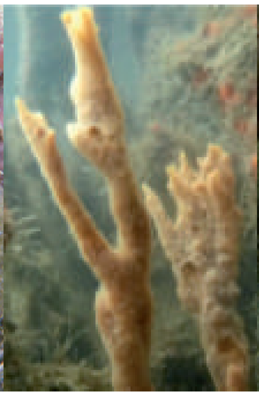

Mycale angulosa
crustose to ramose, bluish orange cream, oscules 1-10 mm

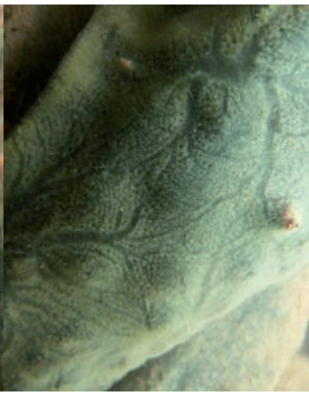

Mycale carmigropila
thin to thick crust, light green to bluish, oscules 2-10 mm, with canals

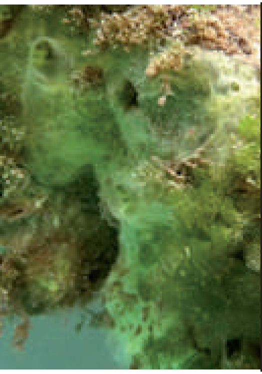

Mycale citrina
small crust, lemon yellow, oscules 1-3 mm

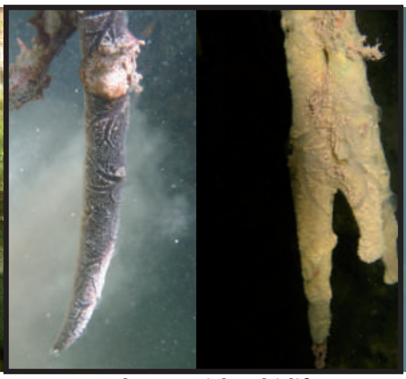

Mycale magnirhaphidifera
thin crust, purple yellow to bluish, oscules 1-3 mm

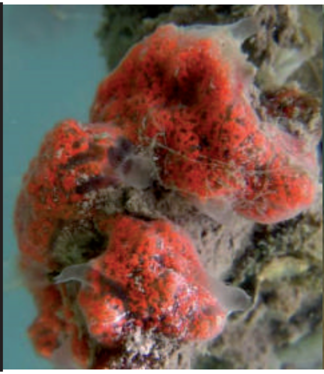

Mycale microsigmatosa
crustose thin to thick, red orange gray, bright red dots, oscules 3-5 mm

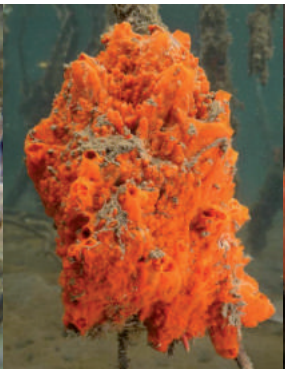

Tedania ignis-fire sponge
massive amorphous , red-orange, oscules 0.5-3 cm

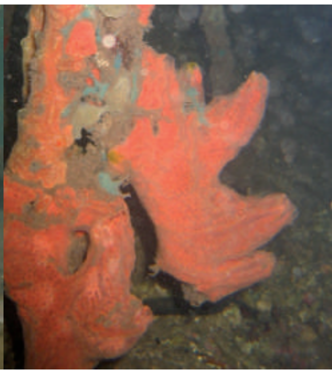

Tedania klausii
massive lobated, red-orange white tinges, oscules 1-3 cm

Scopalinida Order

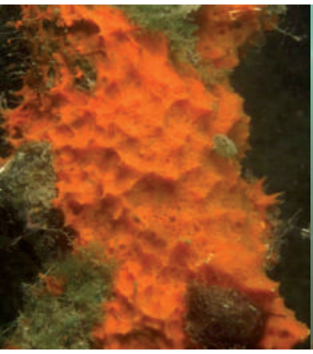

Scopalina ruetzleri
bright to drab orange, soft, conulose, oscules 2-6 mm

Suberitida Order

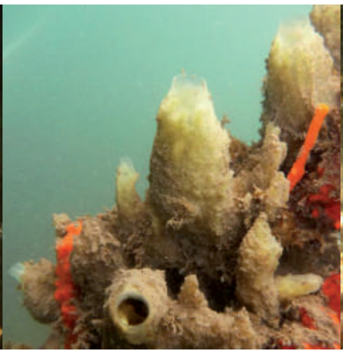

Halichondria magniconulosa
massive, yellowish, rugose, oscules projections 5-10 mm wide

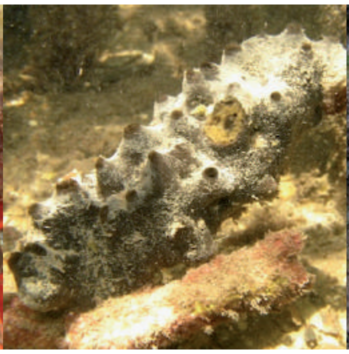

Halichondria melanadocia
dark grey to black out and orange in, oscules 5-10 mm

Tetractinellida Order

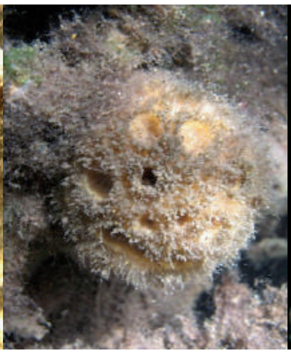

Cinachyrella apion
round, yellowish, porocalices up to 3 mm

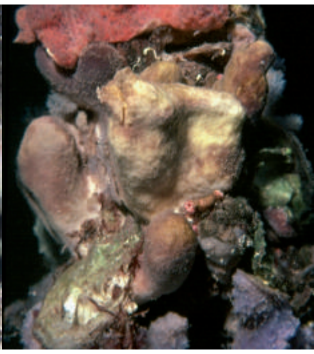

Geodia papyracea
massive brown, oscules 2-5 mm, aggregated on plates
